# Characterization of two bacterial tyrosinases from the halophilic bacterium *Hahella* sp. CCB MM4 relevant for phenolic compounds oxidation in wetlands

**DOI:** 10.1002/2211-5463.13906

**Published:** 2024-10-09

**Authors:** Gustavo de Almeida Santos, Andrea N. B. Englund, Eirin L. Dalleywater, Åsmund Kjendseth Røhr

**Affiliations:** ^1^ Faculty of Chemistry, Biotechnology and Food Science NMBU – Norwegian University of Life Sciences Ås Norway

**Keywords:** *Hahella*, latch mechanism, LID, salinity tolerance, self‐cleavage, tyrosinase

## Abstract

Tyrosinases (TYRs) are type‐3 copper proteins that are widely distributed in nature. They can hydroxylate and oxidize phenolic molecules and are mostly known for producing melanins that confer protection against photo induced damage. TYRs are also thought to play an important role in the ‘latch mechanism’, where high concentrations of phenolic compounds inhibit oxidative decomposition of organic biomass and subsequent CO_2_ release, especially relevant in wetland environments. In the present study, we describe two TYRs, *Hc*Tyr1 and *Hc*Tyr2, from halophilic bacterium *Hahella* sp. CCB MM4 previously isolated at Matang mangrove forest in Perak, Malaysia. The structure of *Hc*Tyr1 was determined by X‐ray crystallography at a resolution of 1.9 Å and represents an uncharacterized group of prokaryotic TYRs as demonstrated by a sequence similarity network analysis. The genes encoding the enzymes were cloned, expressed, purified and thoroughly characterized by biochemical methods. *Hc*Tyr1 was able to self‐cleave its lid‐domain (LID) in a protease independent manner, whereas the LID of *Hc*Tyr2 was essential for activity and stability. Both enzymes showed variable activity in the presence of different metals, surfactants and NaCl, and were able to oxidize lignin constituents. The high salinity tolerance of *Hc*Tyr1 indicates that the enzyme can be an efficient catalyst in the habitat of the host.

## Graphical abstract

Drying marshes, swamps and peatlands comprise wetland environments and huge carbon deposits where organic matter rich in phenolic compounds accumulates. If these environments dry and are exposed to atmospheric oxygen, TYR activity will increase, breaking down the accumulated phenolic compounds. As a result, aerobic respiration and hydrolytic processes are enhanced, leading to higher CO_2_ emissions into the atmosphere.

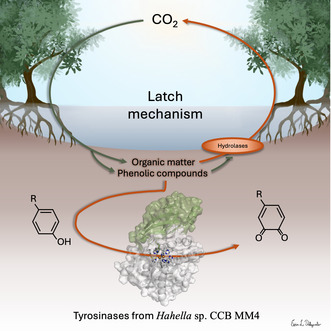

**Abbreviations**
CO_2_
: carbon dioxide
l‐DOPA: l‐3,4‐dihydroxyphenylalanine
LB: Luria–Bertani
MBTH: 3‐methyl‐2‐benzothiazolinone hydrazone
MES: (2‐(N‐morpholino)ethanesulfonic acid)
MOPS: (3‐(N‐morpholino)propanesulfonic acid)
PEG: poly(ethylene glycol)
PPO: polyphenol oxidase
RCSB: Research Collaboratory for Structural Bioinformatics
SSN: sequence similarity network
TYR: tyrosinase

Polyphenol oxidases (PPOs) and tyrosinases (TYRs) are type‐3 copper proteins that are widely distributed in nature, being present in animals, plants, fungi and microorganisms [1, 2, 3, 4]. PPOs and TYRs are vital for vertebrates, providing them invaluable UV‐light protection through melanin biosynthesis [5]. In plants and many invertebrates, TYRs are important in defence mechanisms such as primary immune responses and wound healing [6, 7, 8]. In bacteria, the TYR‐produced melanin can protect DNA from UV‐light‐induced damage, and the activity of TYRs has been associated with virulence of pathogenic microbes [9].

TYRs depend on molecular oxygen to catalyse two different reactions: (a) the ortho‐hydroxylation of monophenols to *o*‐diphenols (monophenolase/cresolase activity) and (b) the oxidation of *o‐*diphenols to *o‐*quinones (diphenolase/catecholase activity) (Fig. 1). Some of these reaction products can spontaneously react and polymerize into different forms of melanin. The role of prokaryotic TYRs in the global carbon cycle has been proposed in the context of the ‘latch mechanism’ hypothesis, which applies to high‐capacity carbon deposits such as drying marshes, swamps and peatlands [10, 11]. These wetland environments (a) accumulate massive quantities of organic material (400–600 gigatonnes of carbon) [12, 13] rich in phenolic compounds that are naturally inhibiting microorganism growth and hydrolytic enzymes [14] and (b) are highly populated by bacteria producing TYRs [15]. In the event of these wetland environments drying, oxygen will inevitably penetrate and lead to increased TYR activity, which will lead to depletion of the accumulated phenolic compounds, triggering aerobic respiration, hydrolytic activity and the potential release of CO_2_ into the atmosphere [10, 14, 16, 17]. The TYRs present in wetland environments originate from secreted enzymes as well as from bacterial lysis [15]. Only a few bacterial TYRs have been structurally characterized according to the UniProtKB/Swiss‐Prot database (https://www.expasy.org/resources/uniprotkb-swiss-prot) and Protein Data Bank (PDB) (https://www.wwpdb.org) and even fewer coming from wetland environments [18].

**Fig. 1:**
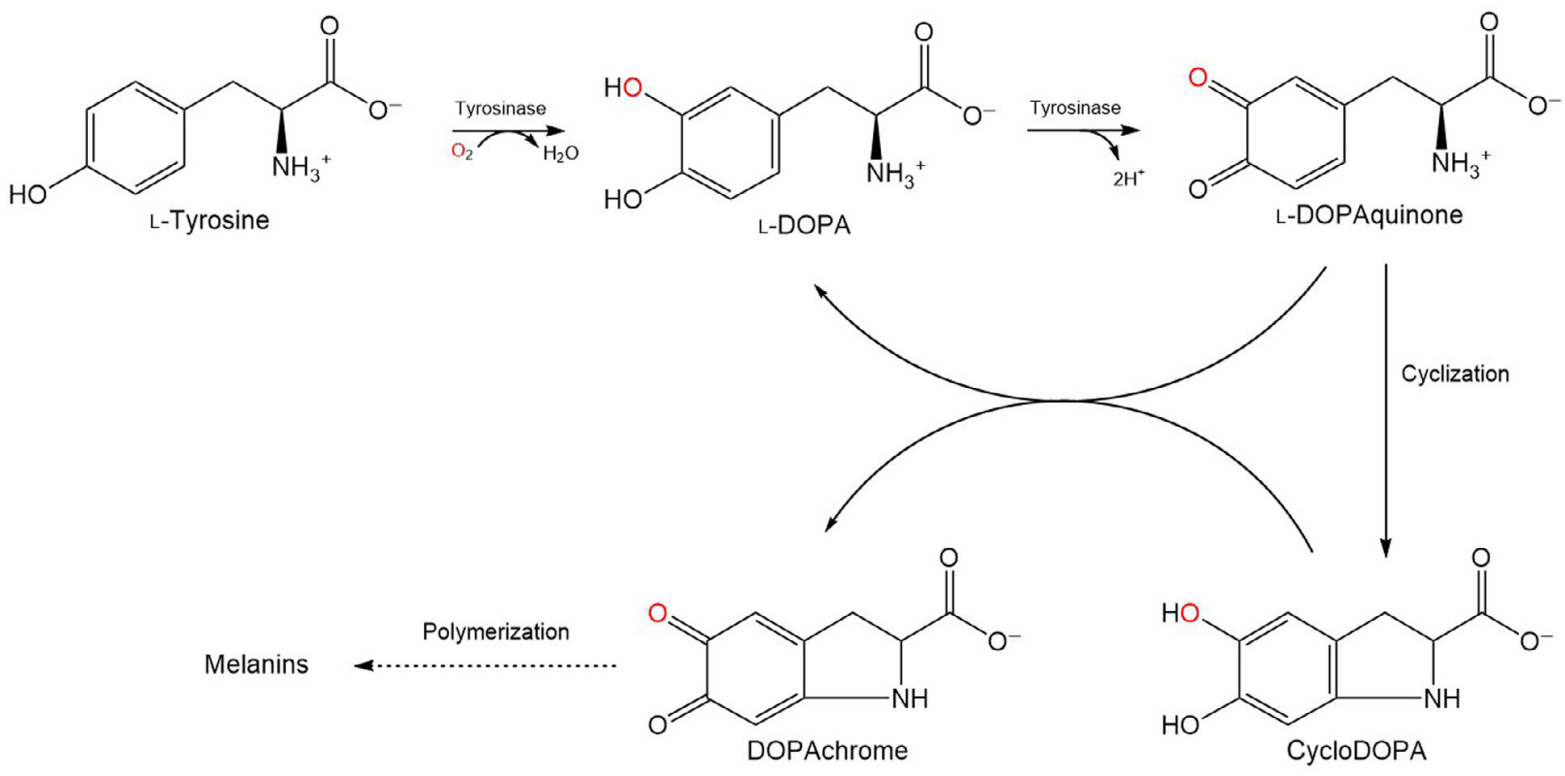
The simplified conversion of tyrosine to l‐DOPA and then to l‐DOPAquinone by TYRs in the presence of oxygen. The spontaneous cyclization to cycloDOPA (also known as leukodopachrome) and its conversion to dopachrome, at the same time as regenerating l‐DOPAquinone to l‐DOPA in the process is also shown. Prepared in chemdraw, version 19.0 (Revvity, Inc., Waltham, MA, USA). Adapted from Otávio de Faria *et al*. (2007) and Solomon *et al*. (2014) [19, 20].

Wetland environments have been bioprospected in search of microbes, their natural products, and secondary metabolites for biotechnological and medical applications [21, 22, 23]. The halophilic gammaproteobacterium *Hahella* sp. CCB MM4 was isolated in 2014 at the estuarine Matang mangrove forest in Perak, Malaysia [24]. This wetland environment experiences tidal variations, large differences in salinity and detritus buildup containing microbes that can degrade complex plant carbohydrates [25].

In the present study, the two TYRs annotated from the *Hahella* sp. CCB MM4 genome [24] were identified and assigned to different groups of prokaryotic TYR genes based on a sequence similarity network analysis. These two genes, coding the proteins *Hc*Tyr1 and *Hc*Tyr2, were cloned, expressed and purified followed by thorough characterization that mapped their substrate profile towards lignin constituents, the effect of inhibitors and enzyme kinetics parameters. Additionally, their activity as a function of pH and temperature, as well as their irreversible inactivation by temperature and pH, were examined. The crystal structure of *Hc*Tyr1 was determined, and a LID‐domain resembling those found in eukaryotic TYRs was found in close interaction with the catalytic domain. Altogether, we present functional and structural data for TYRs known to originate from wetland bacteria.

## Materials and methods

### Materials

All chemicals were purchased from Sigma‐Aldrich (Hamburg, Germany) or TCI (Eschborn, Germany) unless stated otherwise. Salt‐free oligonucleotides were obtained from Thermo Fisher Scientific (Waltham, MA, USA) and DNA and protein concentrations were routinely determined using a NanoDrop One (Thermo Fisher Scientific). Plasmid DNAs were extracted using the E.Z.N.A.® Plasmid DNA Mini Kit (Omega Bio‐tek, Norcross, GA, USA) and sent for sequencing at Eurofins Genomics (Ebersberg, Germany). Figures were prepared using sigmaplot, version 14.0 (Systat Software, Inc., San Jose, CA, USA) and pymol, version 2.4.1 (Schrödinger, Inc., New York, NY, USA) unless otherwise stated.

### Sequence similarity network analysis

A sequence similarity network (SSN) was generated and analysed to provide an overview of bacterial type‐3 copper proteins. The dataset for the SSN analysis consisted of all bacterial enzymes in the InterPro superfamily ‘Di‐copper centre‐containing domain superfamily’ (IPR008922) in the UniProt database (https://www.uniprot.org). The web‐tool Enzyme Function Initiatives Enzyme similarity network (EFI‐ESN) [26] was used to generate the SSN and cytoscape, version 3.9.1 (Cytoscape Consortium, San Diego, CA, USA) [27] was used to visualize it. An initial alignment score threshold of 35 was used to cluster the nodes at the EFI‐EST site (https://efi.igb.illinois.edu/efi-est/), and this was later manually adjusted to 45 in cytoscape. For each cluster in the SSN, multiple sequence alignments, a residue conservation table and weblogos [28, 29] were generated. The py4cytoscape library, version 0.0.11 (https://py4cytoscape.readthedocs.io/en/0.0.11), cyrest [30], clustermaker [31] and autoannotator [32] were used to make the figure.

### Strains, plasmids and target genes

The *Hahella* sp. CCB MM4 genome was previously sequenced, annotated by the NCBI Prokaryotic Genome Annotation Pipeline (Acc. n.: MRYI01000003.1) [24] and two putative TYRs were identified by automated gene prediction by computational analysis, UniProtKB A0A261GRE4 (466 amino acids) and UniProtKB A0A261GVB1 (550 amino acids), named here *Hc*Tyr1 and *Hc*Tyr2, respectively. Both genes encoding *Hc*Tyr1 and *Hc*Tyr2 were cloned in pET22b with a C‐terminal His•Tag®, and expression achieved using *Escherichia coli* BL21 (DE3) cells from NEB (New England Biolabs, Frankfurt, Germany).

### PCR

Sequence‐verified pET22b plasmids containing *Hc*Tyr1 and *Hc*Tyr2 genes were obtained from Genescript (GenScript Biotech B.V., Leiden, The Netherlands) and were used to transform chemically competent *E. coli* BL21(DE3) cells (New England Biolabs) by heat‐shock in accordance with the manufacturer's recommendations. The resulting transformants were then selected on Luria–Bertani (LB) agar plates (10 g·L^−1^ tryptone, 5 g·L^−1^, 5 g·L^−1^ NaCl and 15 g·L^−1^ agar‐agar) supplemented with 100 μg·mL^−1^ ampicillin and the presence of the expression vector was confirmed by colony PCR using Red Taq DNA Polymerase (VWR Life Sciences, Radnor, PA, USA) [33]. To remove the lid‐domain (also known as LID or CAP) and truncate *Hc*Tyr2, an inverse PCR was performed by adapting the protocol by Silva *et al*. [34] and following the recommended protocol for the Q5® High‐Fidelity polymerase (New England Biolabs). All PCR products were analysed by performing a 1% (w/v) agarose gel electrophoresis.

### Expression of recombinant TYR *Hc*Tyr1 and *Hc*Tyr2

The positive transformants selected above were grown overnight (200 r.p.m. at 37 °C for 16 h) in 5 mL of LB (10 g·L^−1^ tryptone, 5 g·L^−1^ yeast extract and 5 g·L^−1^ NaCl) supplemented with 100 μg·mL^−1^ ampicillin. These cultures were then concentrated to 1 mL by centrifugation at 10 000 ***g*** (at room temperature for 1 min), mixed with an equal volume of 50% (v/v) glycerol and stored at −80 °C until further use. Those cryo‐stocks served as an invariable source of inoculum for the subsequent expression cultures. Starter cultures were cultivated in 20 mL of LB supplemented with 100 μg·mL^−1^ ampicillin overnight (200 r.p.m. at 37 °C for 16 h ) and were used to inoculate the main expression cultures in glass bottles filled with 500 mL of terrific broth media (24 g·L^−1^ yeast extract, 12 g·L^−1^ tryptone, 4 g·L^−1^ glycerol, 0.17 mm K_2_HPO_4_ and 0.72 mm KH_2_PO_4_) supplemented 100 μg·mL^−1^ ampicillin to an initial *D*_600_ ≈ 0.1 cm^−1^. The glass bottles were then connected to an air‐pressure system and placed inside a water bath at 37 °C until *D*_600_ was in the range 0.8–1 cm^−1^. At this point, the temperature was lowered to 20 °C and isopropyl thio‐β‐d‐galactoside was added to a final concentration of 0.250 mm to induce protein expression for 16 h. Afterwards, the cells were collected by centrifugation (5500 ***g*** at 4 °C for 25 min). The supernatant was discarded, the cell pellets transferred to 50‐mL tubes and stored at −20 °C until further use. No copper was added during expression.

### Purification and quantification of the recombinant TYRs *Hc*Tyr1 and *Hc*Tyr2

Cell lysates were freshly prepared from frozen cell pellets by resuspension in lysis buffer [0.02 m MOPS, 0.05 m imidazole, 0.5 m NaCl and 5% (v/v) glycerol, pH 7.4] following disruption by sonication for 10 min (10 s ON/10 s OFF cycle), 30% amplitude using a sonicator (VCX‐500; Vibra‐Cell Sonics, Newtown, CT, USA). Lysate clarification was achieved by centrifugation (40 000 ***g*** at 4 °C for 30 min). The clarified lysates were injected into a HisTrap™ prepacked column (Cytiva, Uppsala, Sweden) preequilibrated with lysis buffer. After washing unbound protein with lysis buffer, the purified recombinant TYRs were obtained by eluting with elution buffer (0.02 m MOPS, 0.3 m imidazole, 0.5 m NaCl and 5% (v/v) glycerol, pH 7.4). All steps were performed using an ÅKTA go (Cytiva) at a rate of 3 mL·min^−1^. Further purification was performed using an Amicon Ultra 30K centrifugal filter (Merck Millipore, Darmstadt, Germany) to exchange the elution buffer for storage buffer (0.02 m MOPS and 0.02 m NaCl, pH 7.4) and/or to increase protein concentration. The purified proteins were kept in 0.02 m MOPS and 0.02 m NaCl, pH 7.4, at 4 °C until needed. Protein purity and quantification was routinely performed using SDS/PAGE analysis and NanoDrop One (Thermo Fisher Scientific) spectrophotometer, respectively. No copper was added during purification.

### Enzymatic activity assay and substrate profile

TYR activity was routinely assayed at 25 °C in 96‐well plate format at a wavelength of 475 nm using a Multiskan FC spectrophotometer (Thermo Fisher Scientific). In each well, the reaction contained 205 μL of buffer (0.05 m potassium phosphate buffer, pH 7.0, for *Hc*Tyr1 or 0.05 m sodium citrate buffer, pH 5.5, for *Hc*Tyr2), 20 μL of enzyme (*Hc*Tyr1: 0.02 mg·mL^−1^, *Hc*Tyr2: 0.2 mg·mL^−1^) and 25 μL of substrate (2.5 mm l‐tyrosine, added last to start the reaction). The formation of dopachrome at a wavelength of 475 nm (ε_475_ = 3600 m ^−1^·cm^−1^) was monitored and the enzymatic activities were calculated from the linear part of the absorption vs. time curves (after the lag‐phase but before the subsequent reactions where melanin formation contribute significantly to the absorbance readings). To determine the substrate profile, 200 μL of buffer (0.05 m potassium phosphate buffer, pH 7.0, for *Hc*Tyr1 or 0.05 m citrate buffer, pH 5.5, for *Hc*Tyr2), 25 μL of enzyme (*Hc*Tyr1: 0.05 mg·mL^−1^, *Hc*Tyr2: 0.3 mg·mL^−1^) and 25 μL of 5 mm substrate [dissolved in 20 mm MES, pH 5.5, 25% (v/v) dimethylsulfoxide] were mixed and the activity was evaluated by recording the absorbance spectrum (wavelength 230–600 nm) to detect product formation and/or substrate depletion over time. Before each assay, copper was added at a 2 : 1 molar ratio (2 : 1 Cu(II) : enzyme to ensure a monomer binding two coppers in the active site). To standardize all pipetting steps and reduce human error an Opentrons OT‐2 pipetting robot (Opentrons, Long Island City, NY, USA) was used. All reactions were performed in triplicate and in a 96‐well plate format (unless otherwise stated).

### Enzyme kinetics

Steady‐state kinetic parameters [*V*_max_ and Michaelis constant (*K*_M_)] [35, 36] were determined for l‐tyrosine and l‐3,4‐dihydroxyphenylalanine (l‐DOPA) by non‐linear fitting of Michaelis–Menten plots for *Hc*Tyr1 and *Hc*Tyr2. Absorption curves and spectra were recorded on a Cary UV‐60 spectrophotometer (Shimadzu, Kyoto, Japan) in 1‐cm cuvettes which were maintained at 25 °C using a F25 MH thermostat (Julabo, Seelbach, Germany). As a result of the instability of the TYR reaction products (*o*‐quinones) and their polymerization leading to insoluble compounds (melanins), *o*‐quinones were trapped using the nucleophile 3‐methyl‐2‐benzothiazolinone hydrazone (MBTH). MBTH couples to *o‐*quinones via its amino group generating adducts that remain soluble for longer times and can easily be detected spectrophotometrically at a wavelength of 493 nm (ε_493_ = 29 000 m ^−1^·cm^−1^). It is also important to mention the use of the compound *N*,*N*′‐dimethylformamide in the assay mixture at low concentrations because it renders MBTH soluble and the method can be used over a range of pH values [37, 38]. The kinetic measurements were performed in a total volume of 800 μL containing 656 μL of buffer [0.05 m potassium phosphate buffer, pH 7.0, and 5 mm MBTH, 2% (v/v) *N*,*N*′‐dimethylformamide for *Hc*Tyr1 or 0.05 m sodium citrate buffer, pH 5.5, and 5 mm MBTH, 2% (v/v) *N*,*N*′‐dimethylformamide for *Hc*Tyr2], 80 μL of substrate at different concentrations and 64 μL of enzyme (*Hc*Tyr1: 0.01 mg·mL^−1^ and *Hc*Tyr2: 0.1 mg·mL^−1^). Before each assay, copper was added at a 2 : 1 molar ratio (2 : 1 Cu(II) : enzyme to ensure a monomer binding two coppers in the active site). Control measurements were carried out without enzyme addition to correct for any substrate chemical auto‐oxidation and all measurements were performed in triplicate.

### Characterization of the purified TYRs under different conditions

#### Effect of pH on TYR activity and stability

To determine the TYR activity as a function of pH, 20 μL of enzyme (*Hc*Tyr1: 0.02 mg·mL^−1^ and *Hc*Tyr2: 0.5 mg·mL^−1^) were mixed with 205 μL of a ‘universal’ buffer at different pH values (5 mm sodium citrate, 5 mm potassium phosphate, 5 mm Tris–HCl, 5 mm glycine‐NaOH and 5 mm Na_3_PO_4_‐NaOH) and 25 μL of substrate (2.5 mm l‐tyrosine, added last to start the reaction). The residual activities were determined as described above in the section on ‘Enzymatic activity assay and substrate profile’ and the activity at their optimal pH was taken as 100%. Additionally, the extent of the effect of pH on TYR stability was also assessed by mixing 10 μL of TYR (*Hc*Tyr1: 0.2 mg·mL^−1^ and *Hc*Tyr2: 2 mg·mL^−1^) with 90 μL of ‘universal’ buffer at different pH values and allowing them to incubate at 4 °C for 2, 24, 48 and 120 h. At each data point, 10 μL of incubated enzyme were taken, mixed with 90 μL of 0.02 m MOPS (pH 7.0) and incubated at 4 °C for 1 h before the residual activity was determined as described above in the sectin on ‘Enzymatic activity assay and substrate profile’. The control sample without the treatment was taken as 100%.

#### Effect of temperature on TYR activity and stability

The determination of activity as a function of temperature was evaluated by monitoring the formation of dopachrome at a wavelength of 475 nm (ε_475_ = 3600 m ^−1^·cm^−1^) in cuvettes using a Cary UV‐60 spectrophotometer configurated with multicuvette tray connected to a controlled temperature water bath (Polystat 36 R3; Thermo Fisher Scientific). Briefly, 656 μL of buffer (0.05 m potassium phosphate buffer, pH 7.0, for *Hc*Tyr1 or 0.05 m sodium citrate buffer, pH 5.5, for *Hc*Tyr2) and 80 μL of 2.5 mm tyrosine were incubated until they reached the set temperature (ranging 8–72 °C). Afterwards, and once the temperature stabilized, 64 μL of enzyme (0.01–0.1 mg·mL^−1^) was added when the formation of dopachrome was being monitored at a wavelength of 475 nm. The enzymatic activities were calculated from the linear part of the absorption–time curves (after the lag‐phase but before the subsequent reactions where melanin contribute significantly) and their activity at the highest rate was taken as 100%.

The melting temperature curve was determined using a StepOnePlus real time PCR machine (Thermo Fisher Scientific) and SYPRO™ Orange protein stain (λ_excitation_ 492 nm, λ_emission_ 585 nm; Thermo Fisher Scientific). Briefly, 50 μL of protein at 60 μm was mixed with 12.5 μL of SYPRO™ Orange (8×) and 37.5 μL of buffer (0.02 m MOPS, pH 7.0) and then subjected to a temperature ramp (8 °C for 10 min, 2 °C·min^−1^ up to 99 °C). To determine the fraction of the unfolded enzyme (*f*_U_) at each temperature, the relative fluorescence intensity was normalized using:

(1) $$f_{U}=\frac{y_{F}-y}{y_{F}-y_{U}}$$

where *y* is the fluorescence intensity measured at each temperature data point (*T*) and *y*_F_ and *y*_U_ are the maximum and minimum of relative fluorescence for the folded and unfolded state, respectively, at each *T*. The normalized data was then fitted to a sigmoidal tendency equation [Eqn (2)], using sigmaplot, version 14.0 (Systat Software, Inc.), where *a* is the maximum percentage of the unfolded fraction (approximately 100), *b* is the slope, *T* is the temperature and *T*_m_ is the melting temperature to be determined by solving the equation.

(2) $$f_{U}=\frac{a}{1+e^{\frac{T-T_{m}}{b}}}$$

Additionally, to evaluate the reversible/irreversible effect of temperature on TYR stability, the enzymes were incubated in 0.02 m MOPS (pH 7.0) at 4, 25, 40 and 60 °C and data collected at 0, 5, 30 and 60 min. At each data point a sample of incubated enzyme was taken and placed on ice for 1 h before the residual activity was determined as described above in the section on ‘Enzymatic activity assay and substrate profile’. A sample without the treatment was taken as 100%.

#### Influence of effectors on TYR activity

To investigate the influence of metal ions (Li^1+^, Ni^2+^, Co^2+^, Cu^2+^, Ca^2+^, Mn^2+^ Mg^2+^, Al^3+^ and Fe^3+^), chemical reagents (EDTA, dithiothreitol, Triton X‐100 and SDS) and salinity (as sodium chloride) on the activity of *Hc*Tyr1 and *Hc*Tyr2, the relative activity was evaluated by adding 1 or 5 mm of metal ions, 0.5, 1 and 2 m of the chemical reagents (Triton X‐100: 0.05%, 0.1% and 0.2% (v/v)), and 0.1–5 m of sodium chloride. The activity without adding the effectors was taken as 100%. Furthermore, to reduce the influence of metal ions complexation by the ‘universal’ buffer and improve metal ion solubility, 0.02 m MES (pH 5.5) was used instead. The residual activity was determined as described above in the section on ‘Enzymatic activity assay and substrate profile’.

### Protein crystallization, X‐ray diffraction and model building

Conditions for the growth of *Hc*Tyr1 and *Hc*Tyr2 crystals were refined based on an initial hit obtained with 0.2 m LiSO_4_, 0.1 m Bis‐Tris, pH 5.5, and 20% (w/v) poly(ethylene glycol) (PEG) 4000 for *Hc*Tyr1 and 0.2 m sodium citrate tribasic dihydrate and 20% (w/v) PEG 3350 for *Hc*Tyr2 in a sitting drop vapour diffusion 96‐well 2‐Drop Plates set‐up (Molecular Dimensions, Sheffield, UK). Single crystals suitable for crystallography grew over the course of 1–4 weeks in four by six‐well plates using the hanging drop vapour diffusion set‐up: 2.2‐μL drops consisting of 1.1 μL of purified enzyme in 0.02 m MOPS, pH 7.0 (*Hc*Tyr1: 13 ± 1 g·L^−1^; *Hc*Tyr2: 10 ± 1 g·L^−1^) mixed with 1.1 μL of reservoir solution [0.2 m LiSO_4_, 0.1 m Bis‐Tris, pH 5.5, and 20% (w/v) PEG 4000 for *Hc*Tyr1; 0.2  sodium citrate tribasic dihydrate and 20% (w/v) PEG 3350 for *Hc*Tyr2]. Both set‐ups were equilibrated via vapour diffusion with 600 μL of the respective reservoir solution at room temperature and protected from light. Crystals were harvested using MiTeGen® loops (Ithaca, NY, USA), soaked with cryo‐protectant (50% (w/v) glucose) and plunged into liquid nitrogen where they remained until the diffraction experiment. Data were collected at beamline ID‐23 and ID‐30 at the European Synchrotron Radiation Facility (ESRF, Grenoble, France) at 100 K, wavelength of 0.873 Å (14.2 keV) using the PILATUS detector. All data were indexed, integrated, scaled and merged using autoPROC [39]. The structure of *Hc*Tyr1 in crystals was solved by molecular replacement using phaser [40] and the respective Alphafold2 model. Refinement was performed using refmac5 [41]. Manual model building, real‐space refinement and structure validation were performed using coot [42]. The coordinates and structure factors of *Hc*Tyr1 have been deposited in the Research Collaboratory for Structural Bioinformatics (RCSB) PDB under accession code 8B74. The structures of *Hc*Tyr1 and *Hc*Tyr2 were also predicted using Alphafold2 [43].

## Results and Discussion

### *Hc*Tyr1 and *Hc*Tyr2 sequence analysis

**Fig. 2:**
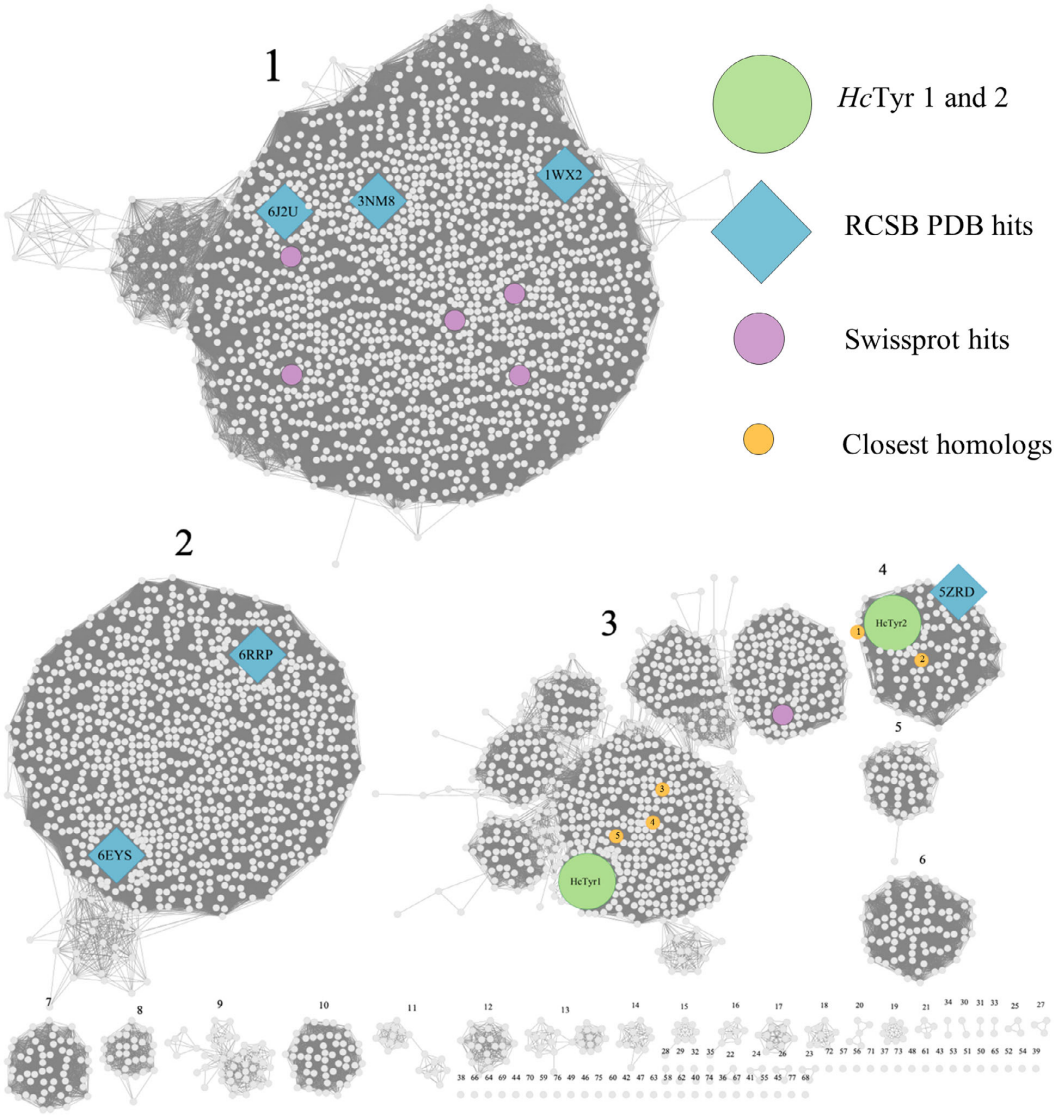
The SSN analysis revealed 4223 nodes and 1 518 020 edges. Only six proteins are annotated in the Swiss‐Prot database (purple circles) and other six have their structure elucidated (RCSB PDB database, blue diamonds). *Hc*Tyr1 and *Hc*Tyr2, are in clusters 3 and 4, respectively (green circles).

The SSN analysis (Fig. 2) based on all bacterial sequences in the IPR008922 family revealed that the two subjects of this study, *Hc*Tyr1 (UniProtKB A0A261GRE4, 466 amino acids) and *Hc*Tyr2 (UniProtKB A0A261GVB1, 550 amino acids), are found in cluster numbers 3 and 4, respectively. Furthermore, the SSN analysis also revealed 4223 unique nodes (each corresponding to a type‐3 copper protein) of which only six are annotated in the Swiss‐Prot database and another six have their crystal structure elucidated (RCSB PDB database). Thus, the sequence space of bacterial TYRs remains experimentally unexplored.

The *Hc*Tyr1 and *Hc*Tyr2 proteins have theoretical molecular weights of 52 and 61 kDa respectively, and 25.7% amino acid sequence identity between them (Uniprot Align). The two *Hc* TYRs have relatively low similarity (NCBI BLAST; https://blast.ncbi.nlm.nih.gov) to other well characterized TYRs from the genus *Bacillus*, *Bacillus thuringiensis* (*Bt*Tyr) (Q6SS53, 27.0%, 28.1%) and *Bacillus aryabhattai* TCCC 111983 (*Ba*Tyr) (A0A6H1TJ97, 28.3%, 34.8%), *Priestia megaterium* (*Pm*Tyr) (B2ZB02, *Hc*Tyr1: 26.9%, *Hc*Tyr2: 35.7%, formerly classified as *Bacillus megaterium*) [44], *Streptomyces* genus, such as *Streptomyces kathirae* (*Sk*Tyr) (A0A077HD11, 26.8%, 37.3%) and *Streptomyces castaneoglobisporus* (Q83WS2, 30.1%, 31.9%), and others, such as bacteria *Marinomonas mediterranea* (Q5VM57, 44.5%, 33.9%), and fungi *Aspergillus niger* ATCC 1015 (G3XLG7, 23.3%, 20.7%) and *Agaricus bisporus* (C7FF05, 25.9%, 26.2%).

### Protein purification

The expression and purification of *Hc*Tyr1 and *Hc*Tyr2, as described in the Materials and methods, yielded around 100–125 mg of fusion protein per litre of medium after capture and purification. Removal of the elution buffer using Amicon® centrifugal allowed recovery yields between 80% and 100% corresponding to approximately 80–100 mg of recombinant protein per litre of expression culture. The molecular weight of purified *Hc*Tyr1 and *Hc*Tyr2 was confirmed by SDS/PAGE (Fig. S1) and was approximately 52 and 61 kDa, respectively, which is identical to the theoretical molecular weight (ProtParam, Expasy online tool; https://web.expasy.org/protparam).

### Self‐processing *Hc*Tyr1 lid domain

**Fig. 3:**
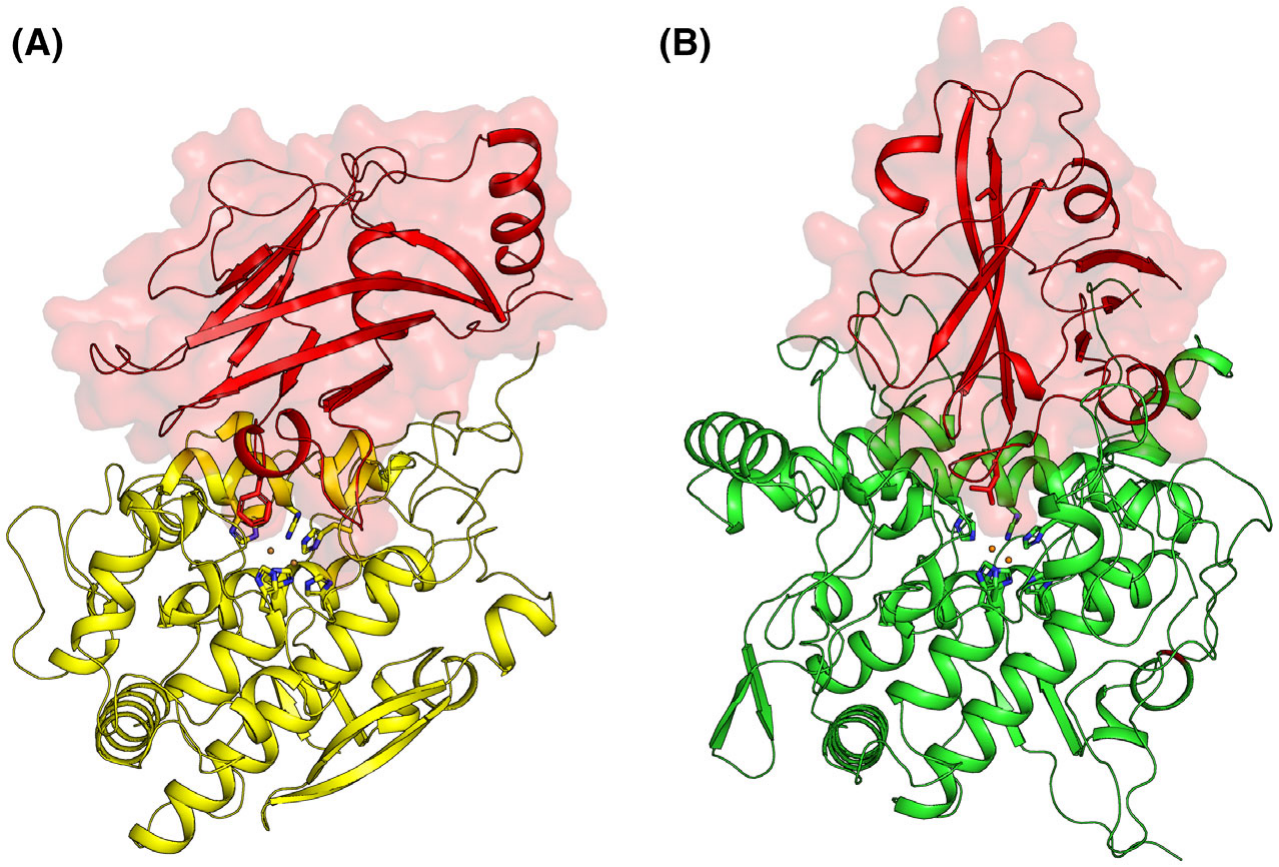
The cartoon representation of the Alphafold2 models *Hc*Tyr1 (A) and *Hc*Tyr2 (B). The LID‐domains are coloured in red, and the placeholder residue is represented as a stick in the same colour; TYR‐domain of *Hc*Tyr1 is coloured in yellow and TYR‐domain of *Hc*Tyr2 is coloured in green. Copper atoms in the active site (placed by superposition with PDBid 3NM8 to visualize the active site) are shown as spheres in orange and the histidines coordinating them are shown as sticks.

Most TYRs either (a) do not have a lid‐domain (also known as LID or CAP), such as *Pm*Tyr from *P. megaterium* [45], *Ba*Tyr from *B. aryabhattai* [46] and *Bt*Tyr from *Burkholderia thailandensis* [47], or (b) have a LID that require external proteolytic cleavage such as *Ab*PPO4 from *A. bisporus*, *Md*PPO1 from *Malus domestica* or *Tr*Tyr2 from *Trichoderma reesei* [48, 49, 50, 51]. *Hc*Tyr1 and *Hc*Tyr2 both have a LID overlaying their active site on the TYR‐domain (TYR), each with a different placeholder residue, Phe397 and Leu469, respectively (Fig. 3). Although the LID remained attached to *Hc*Tyr2, for *Hc*Tyr1, it was slowly self‐processed during storage (> 2‐month period at 4 °C following purification), resulting in a form that was approximately 10‐fold more active; henceforth named cleaved‐*Hc*Tyr1 (Fig. S2). Latent‐*Hc*Tyr1 and cleaved‐*Hc*Tyr1 were used as shown in Fig. S2, without further purification. To investigate the causes for this ‘spontaneous’ LID self‐processing in *Hc*Tyr1, we tested the influence of pH (Fig. S3), addition of a mix of transition metal ions (Co^2+^, Cu^2+^, Fe^3+^, Mn^2+^, Ni^2+^ and Zn^2+^) (Fig. S4), addition of Cu^2+^ alone (Fig. S5), and temperature influence and presence of a protease inhibitor (Fig. S6), although none induced LID cleavage to significant levels in the time period tested here. The activation mechanism of *Hc*Tyr1 is likely dependent on a specific amino acid sequence capable of arranging itself to cleave a nearby peptide bond, such as in the previously reported studies of a TYR‐induced self‐cleavage reaction (protease independent) in *Md*PPO1 from *M. domestica* [50] and *Pa*PPO from *Prunus armeniaca* [52]. We have also cloned and expressed a truncated version of *Hc*Tyr2 (residues 1–425, no LID) and assessed its activity aiming to determine whether the absence of the LID in *Hc*Tyr2 would also result in a more active version. However, the truncated *Hc*Tyr2 exhibited marginal activity and started to precipitate shortly after purification, indicating that the LID of *Hc*Tyr2 is important for both stability and activity.

### Effects of pH on activity and stability

**Fig. 4:**
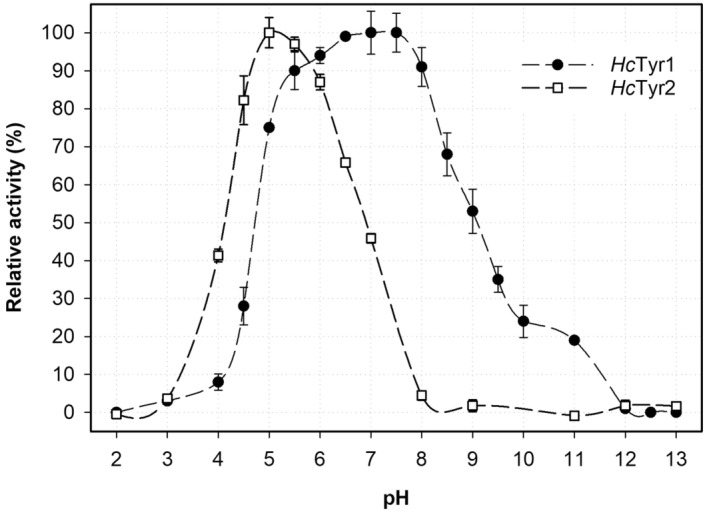
The relative activity of cleaved‐*Hc*Tyr1 (full circles) and *Hc*Tyr2 (empty squares) at different pH values ranging from 2 to 13. The error bars represent 1 SD of the mean from three replicates. SD, standard deviation

The activity and stability as a function of pH for *Hc*Tyr1 and *Hc*Tyr2 was assessed at different pH values from 2 to 13. As shown in Fig. 4, *Hc*Tyr1 displayed maximum activity between pH 6 and 8, which is observed for other bacterial TYRs *Pm*Tyr from *P. megaterium* (pH 7.0) [45], *Pseudomonas putida* F6 (*Pp*Tyr, pH 7.0) [53], *Rhizobium etli* CFN42 (*Re*Tyr, pH 7.0) [54] and fungal TYR from *Pycnoporus* strains (pH 6.0–7.0) [55], whereas *Hc*Tyr2 exhibited maximum activity between pH 4.5 and pH 6, similar to *Ba*Tyr from *B. aryabhattai* (pH 5.0) [46] and *Bt*Tyr from *Bu. thailandensis* (pH 5.0) [47]. Furthermore, *Hc*Tyr1 exhibited a relatively broad pH range of activities (> 50% between pH 4.5 and 9.5), whereas *Hc*Tyr2 revealed a rather narrow pH range of activity with > 50% between pH 4 and 7 and no detectable activity above pH 8. The determinants of pH activity in *Hc*Tyr2 appear to be linked to electrostatic charge alterations at the TYR and LID because their isolectric points (pI) are 4.8 and 8.6, respectively, as calculated using IPC webserver [56]. As the pH increases, the TYR charges become more negative, whereas the LID is relatively less affected by pH changes (Fig. S7). This may lead to stronger interactions between the two domains as the pH increases, ultimately resulting in complete enclosure of the active site and inactivation above pH 8. Indeed, a similar case has been reported for *Bt*Tyr [47], which has 64.5% sequence similarity and is structurally similar to *Hc*Tyr2 with an rmsd of just 1.22 Å. By contrast, the interaction nature between the LID and TYR of *Hc*Tyr1 are largely hydrophobic and less electrostatic (Fig. S7) which could help explain the broader pH range of activity. Furthermore, there was no significant change on the activity as a function of pH between latent‐*Hc*Tyr1 and cleaved‐*Hc*Tyr1.

**Fig. 5:**
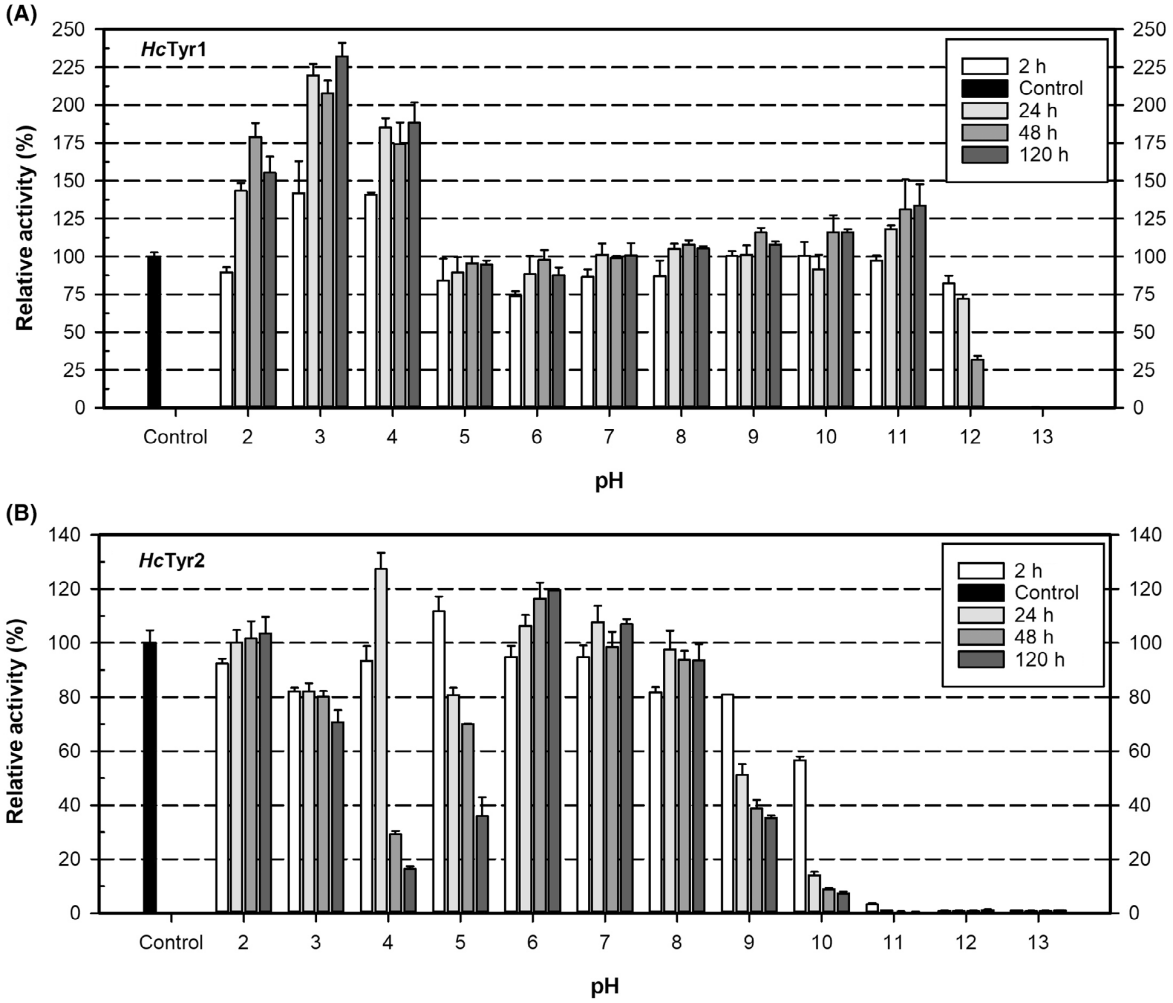
The influence of pH on stability of latent‐*Hc*Tyr1 (A) and *Hc*Tyr2 (B) was analysed by monitoring the residual activity at different pH conditions in ‘universal’ buffer (5 mm sodium citrate, 5 mm potassium phosphate, 5 mm Tris‐HCl, 5 mm glycine‐NaOH and 5 mm Na_3_PO_4_‐NaOH) after incubating the enzyme at 4 °C for 2, 24, 48 and 120 h. The error bars represent one standard of the mean from three replicates.

The pH influence on *Hc*Tyr1 and *Hc*Tyr2 stability was assessed (Fig. 5) and it revealed a rather unexpected behaviour for latent‐*Hc*Tyr1. The incubation at acidic pH values (pH 2 to 4) increased latent‐*Hc*Tyr1 relative activity 1.5 to 2‐fold as the incubation proceeded for 120 h, with this effect being observed as early as 2 h of incubation. Although no loss of activity is seen from pH 2 to 11, pH 12 is irreversibly damaging the enzyme to the point where, after 120 h of incubation, no activity is recorded. Regarding *Hc*Tyr2, incubation at pH 4 and 5 is irreversibly damaging the enzyme over time because its activity is gradually decreasing; however, this effect is likely to be connected to *Hc*Tyr2 pI; theoretical: 5.07 ± 0.34, as calculated using IPC webserver [56]. Furthermore, protein aggregation was observed by the naked eye when adding the enzyme to the incubation solution at pH 4 and 5 during the experimental set‐up (2 mg·mL^−1^ enzyme stock) but not when setting up the pH‐dependence activity assay (0.2 mg·mL^−1^ enzyme stock), revealing that the aggregation observed is dependent on protein concentration. This corroborates that a protein will be less soluble at pH values close to its pI. Alkaline pH values are damaging *Hc*Tyr2 over time as well, starting from pH 9 where, after 120 h of incubation, the enzyme only recovers 40% activity. These results indicate that *Hc*Tyr1 is more stable than *Hc*Tyr2 under acidic, neutral and alkaline pH conditions.

The increased activity of latent‐*Hc*Tyr1 after incubation at acidic pH values was investigated further and the formation of a dimer when the protein is in its holo‐form was observed following SDS/PAGE analysis (Fig. S8). Although this dimer is fully reverted to its monomeric state once exposed to reducing conditions (50 mm dithiothreitol) and heat treated (95 °C for 15 min) (Fig. S9), its oligomeric form is maintained after transferring it to pH 7 (Fig. S10). The increased activity in oligomeric has also been observed (albeit on other protein classes) and it was reported that disrupting this form severely hampers activity [57, 58]. Even though, to our knowledge, the native state of this enzyme is monomeric, the nature of *Hc*Tyr1 aggregation observed at acidic pH values is thought to be a combination of two factors essential for the oligomerization to occur: (a) formation of a disulfide bridge and (b) conformational changes necessary for it to be formed. There are three cysteines present in *Hc*Tyr1, two in the TYR with virtual zero solvent accessible surface area (< 0.4 Å^2^) and one in the LID with a higher accessible surface area of approximately 57 Å^2^ (pymol computed values) (Fig. S11). In addition, Cu^2+^ supplementation not only leads to protein conformation changes (apo‐ to holo‐form), but also catalyses the oxidation of thiols in the presence of molecular oxygen [59], which, when combined with the increased protonation of amino acids occurring at acidic pH values, can lead to sufficient conformational changes to further expose the cysteine in the LID, allowing it to bond with another cysteine from another *Hc*Tyr1 molecule, thus forming a dimer and increasing substrate accessibility to the active site.

Regarding the *in vivo* implications of the pH effect, there are no records of *Hahella* sp. CCB MM4 cytoplasm pH. We assume that it follows the general ‘rules’ of bacterial homeostasis and maintains a pH between 6 and 8 despite the environmental conditions [60]. Thus, *Hc*Tyr1 and *Hc*Tyr2 may not experience pH values below 6 or above 8 in the cytoplasm and both operate close to their optimum pH. However, by contrast, in *ex vivo* conditions if there is leakage as a result of cell lysis, *Hc*Tyr1 and *Hc*Tyr2 will be exposed to pH values lower than 5 [61] leading to increased activity without loss of stability for *Hc*Tyr1. *Hc*Tyr2, on the other hand, will not be as stable as *Hc*Tyr1 as observed *in vitro*.

### Effect of temperature on activity and stability

**Fig. 6:**
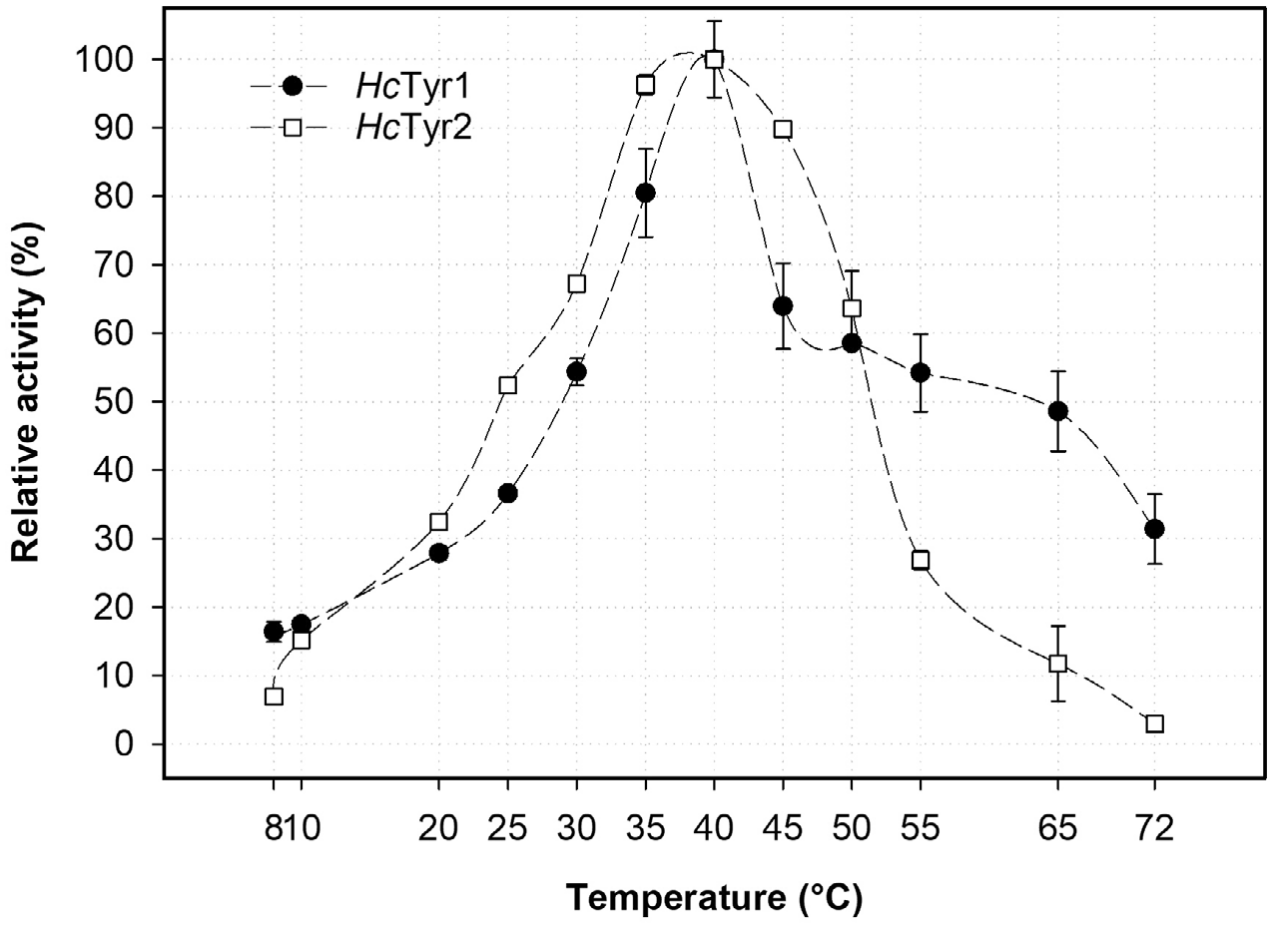
The relative activity cleaved‐*Hc*Tyr1 (full circles) and *Hc*Tyr2 (empty squares) as a function of temperature (8–72 °C) determined at each enzyme's optimum pH. The error bars represent 1 SD of the mean from three replicates. SD, standard deviation

**Fig. 7:**
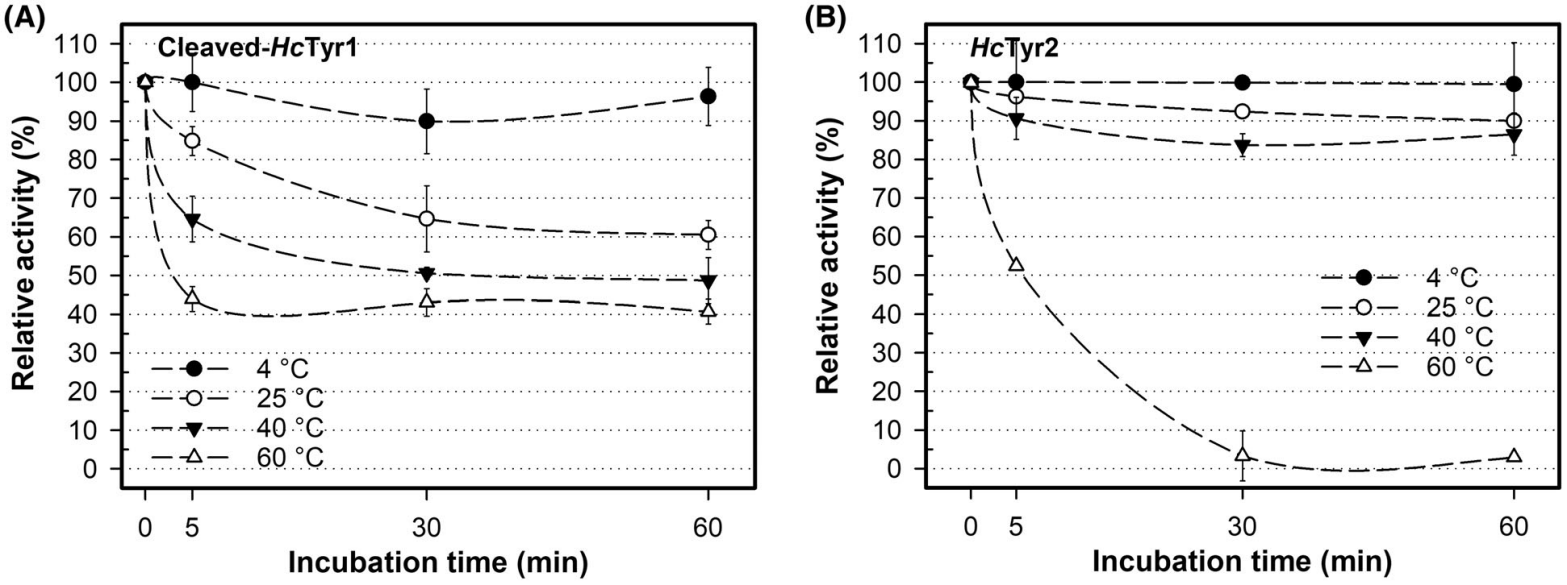
The stability of cleaved‐*Hc*Tyr1 (A) and *Hc*Tyr2 (B) when subjected to temperature over time. The influence of temperature was determined by determining the residual activity at 4, 25, 40 and 60 °C from 0 min to 60 min in 20 mm MOPS (pH 7.0) and then allowed to recover for 60 min on ice. The activity was determined at each enzyme's optimum pH. The error bars represent 1 SD of the mean from three replicates. SD, standard deviation

The activity as a function of temperature for *Hc*Tyr1 and *Hc*Tyr2 was assessed at different temperatures (8–72 °C). As shown in Fig. 6, both *Hc*Tyr1 and *Hc*Tyr2 presented the maximum activity at approximately 40 °C where *Hc*Tyr1 had > 90% relative activity between 35 °C and 45 °C, the activity decreased for both when the temperature was increased from 45 °C to 72 °C or reduced from 35 °C to 8 °C. The optimal temperature for both TYRs is within the range of other typical bacterial TYRs, including *Sk*Tyr from *S. kathirae* (45 °C) [62], *Pp*Tyr from *Ps. putida* (30 °C) [53], *Pm*Tyr from *P. megaterium* (50 °C) [45] and *Re*Tyr from *R. etli* (50 °C) [54], as well as *Polyporus arcularius* fungal TYR (50 °C) [63] to name but a few. Moreover, although *Hc*Tyr1 maintained 60–50% of activity between 40 °C and 65 °C and retained 30% of activity at 72 °C, *Hc*Tyr2 exhibited a typical bell‐shape profile and its activity goes below 50% under 25 °C and past 45 °C and, at 72 °C, the enzyme no longer exhibits activity. Unlike *Pm*Tyr, *Re*Tyr and *Ba*Tyr, which demonstrated a high activity (> 70%) in the temperature range from 40 °C to 70 °C, from 30 °C to 60 °C and from 40 °C to 90 °C, respectively [45, 46, 54], *Hc*Tyr1 and *Hc*Tyr2 do not exhibit the same behaviour and, in comparison, have a narrow range of temperatures (30 °C up to approximately 45 °C) where activity is ≥ 70%. Furthermore, cleaved‐*Hc*Tyr1 is more resistant than *Hc*Tyr2 to higher temperatures (60 °C) and regained 42 ± 3% of activity after 60 min of exposure, whereas *Hc*Tyr2 has negligible activity (< 3%) (Fig. 7). However, *Hc*Tyr2 is less affected by moderate temperatures (20 °C and 40 °C) after 60 min of exposure, recovering ≥ 85% of activity at 20 °C and 40 °C vs. the 60 ± 4% and 49 ± 5% of cleaved‐*Hc*Tyr1.

**Fig. 8:**
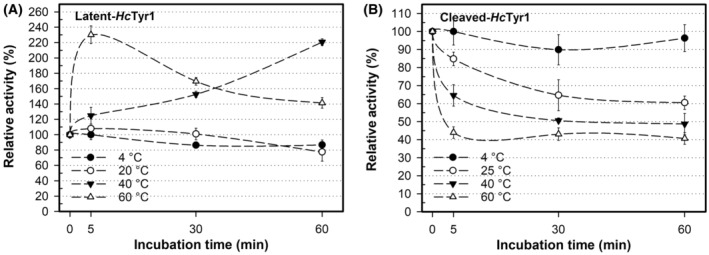
The stability of latent‐*Hc*Tyr1 (A) and cleaved‐*Hc*Tyr1 (B) when subjected to temperature over time. The influence of temperature was determined by determining the residual activity at 4, 25, 40 and 60 °C from 0 min to 60 min in 20 mm MOPS (pH 7.0) and then allowed to recover for 60 min on ice. The activity was determined at each enzyme's optimum pH. The error bars 1 SD of the mean from three replicates. SD, standard deviation

**Fig. 9:**
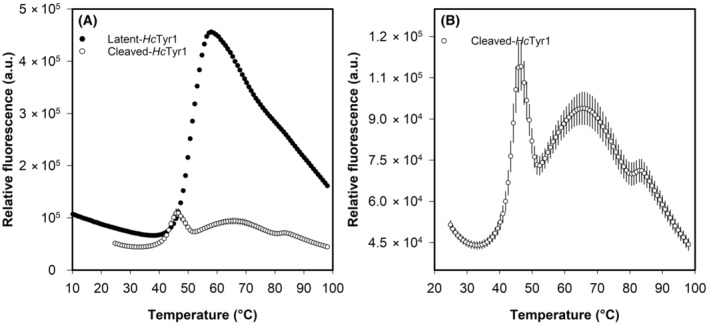
The raw data of the thermal denaturation curves of latent‐*Hc*Tyr1 (full circles) and cleaved‐*Hc*Tyr1 (empty circles). A) the comparison between the thermal denaturation curve of latent‐*Hc*Tyr1 and cleaved‐*Hc*Tyr1, B) the thermal denaturation of cleaved‐*Hc*Tyr1. The error bars represent 1 SD of the mean from three replicates. SD, standard deviation

**Fig. 10:**
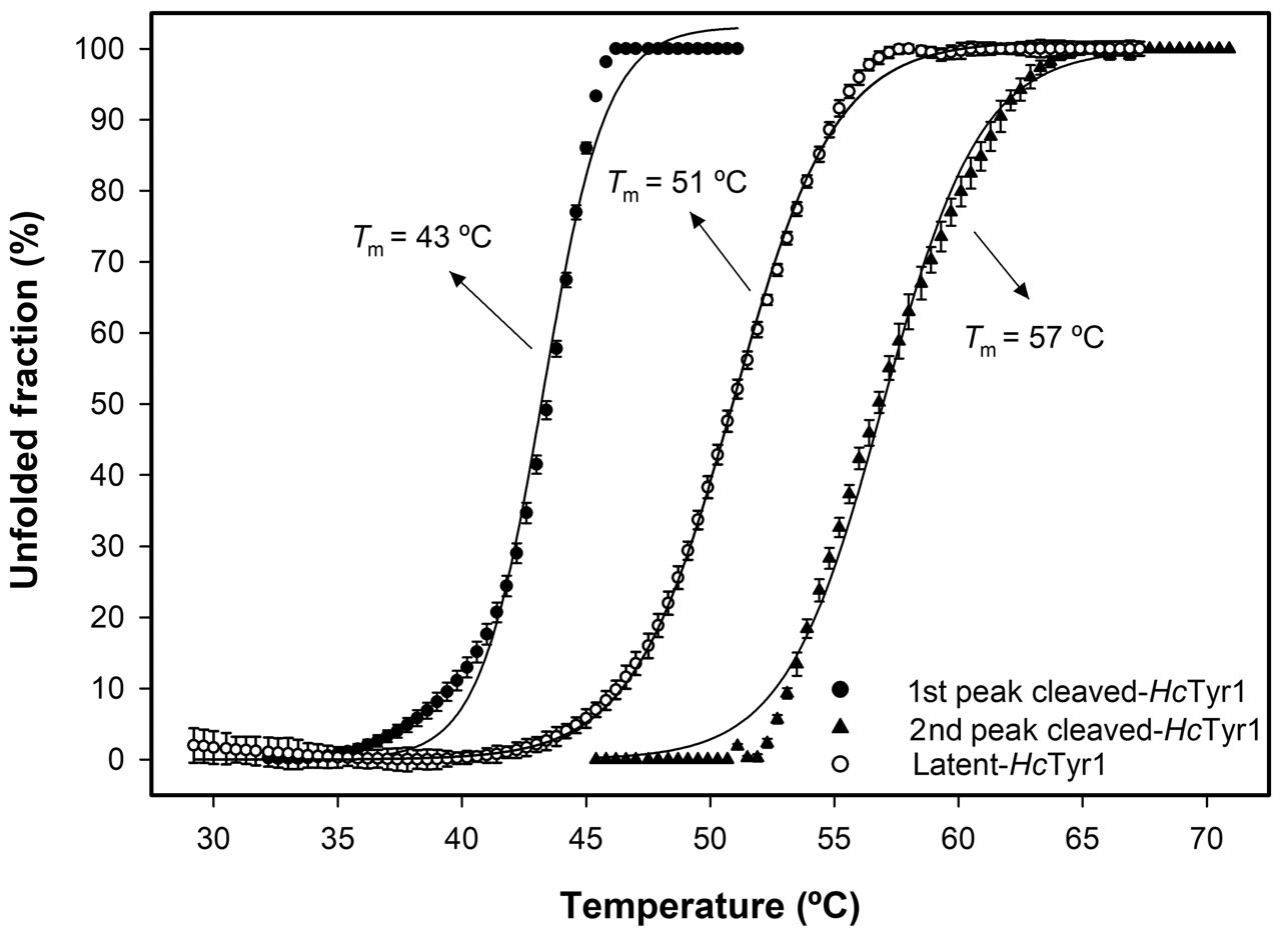
The fitted curve to the normalized thermal denaturation curves of cleaved‐*Hc*Tyr1 (full circles and triangles) and latent‐*Hc*Tyr1 (empty circles). The error bars represent 1 SD of the mean from three replicates. SD, standard deviation

The activation of *Hc*Tyr1 (leading to cleaved‐*Hc*Tyr1) did not alter its activity as a function of temperature but it did affect its stability and how it reacts when subjected to temperature over time (Fig. 8). Indeed, although the activated form of *Hc*Tyr1 is temperature labile, being slowly inactivated over time, latent‐*Hc*tyr1 can cope better at 20 °C with no loss of activity measured. Furthermore, the increased activity measured at 40 °C and 60 °C by latent‐*Hc*Tyr1 can only be attributed to conformational changes that augments active site exposure, thus shortly increasing activity for 5 min at 60 °C and gradually over time at 40 °C. These conformational changes are the steady formation of a dimer over time at 40 °C (Fig. S12) which correlate well with the measured gradual increase in relative activity over time at 40 °C as previously shown in Fig. 8. In hindsight, this dimer formation is not an exclusive behaviour of exposure to acidic pH values, and it appears that the dimer will form whenever the protein is in holo‐form and experiences conformational changes, in this case, because of the temperature increase. Furthermore, the activation of *Hc*Tyr1 changed the thermal denaturation curve as well (Fig. 9). Latent‐*Hc*Tyr1 has a smooth and distinctive transition between the folded and unfolded state when exposed to increasing temperatures (Fig. 9A), whereas cleaved‐*Hc*Tyr1 has relatively low fluorescence and a small pitfall of stability at approximately 52 °C (Fig. 9B). To have a better overview, the two peaks observed at cleaved‐*Hc*Tyr1 thermal denaturation curve were treated independently of each other, originating a first melting temperature (*T*_m_) of 43 °C and a second *T*_m_ of 57 °C. Interestingly, the *T*_m_ of latent‐*Hc*Tyr1 was 51 °C, which is the average of the two calculated melting temperatures of cleaved‐*Hc*Tyr1 (Fig. 10). In addition, the small pitfall of stability observed for cleaved‐*Hc*Tyr1 could help to explain its ability to withstand a temperature of 60 °C for 1 h without completely unfolding and still displaying 40% activity, as shown in Fig. 7.

The *in vivo* implications of these findings are not relevant given that the natural habitat of the host has a mean annual temperature between 23.7 °C and 33.4 °C [64]. Temperatures rarely exceed the 40 °C barrier and thus it is unlikely that the TYRs will undergo thermal denaturation under *in vivo* or *ex vivo* conditions.

### Effects of metal ions, chemical reagents and salinity on TYR activity

Metal ions play a key role in many microorganisms, animals, insects and plants. Their working concentrations in cells are tightly controlled because they can drastically influence enzymatic behaviour [65, 66, 67]. The effect of metal ions, chemical reagents, and salinity on *Hc*Tyr1 and *Hc*Tyr2 activity was investigated as shown in Table 1. In summary, the addition of the tested metal ions produced similar effects on both TYRs with a few exceptions.

**Table 1:** The influence of metal ions, salinity, and chemical compounds on HcTyr1 and HcTyr2. ND, not detected; SD, standard deviation from three replicates.

Metal ions/inhibitors	Concentration (mm)	Residual activity (%), mean ± SD^a^	
		*Hc*Tyr1	*Hc*Tyr2
None	–	100 ± 4	100 ± 4
AlCl_3_	1	71 ± 4	18 ± 2
	5	55 ± 3	14 ± 0
CaCl_2_	1	121 ± 2	105 ± 8
	5	120 ± 4	107 ± 6
CoCl_2_	1	99 ± 3	107 ± 6
	5	95 ± 7	107 ± 2
CuCl_2_	1	33 ± 2	45 ± 1
	5	21 ± 1	28 ± 1
FeCl_3_	1	ND	ND
	5	ND	ND
LiCl	1	111 ± 5	98 ± 8
	5	110 ± 8	93 ± 6
MnCl_2_	1	102 ± 4	100 ± 6
	5	104 ± 1	102 ± 1
MgCl_2_	1	102 ± 1	105 ± 8
	5	106 ± 4	112 ± 2
NiCl_2_	1	103 ± 5	122 ± 8
	5	67 ± 4	90 ± 5
ZnCl_2_	1	29 ± 1	108 ± 6
	5	16 ± 5	93 ± 6
NaCl	100	100 ± 3	100 ± 4
	250	134 ± 8	58 ± 3
	500	121 ± 5	58 ± 2
	1000	103 ± 1	55 ± 3
	2500	101 ± 4	56 ± 2
	5000	89 ± 3	42 ± 3
Dithiothreitol	0.5	ND	ND
	1	ND	ND
	2	ND	ND
EDTA	0.5	115 ± 1	101 ± 1
	1	103 ± 7	105 ± 2
	2	105 ± 2	107 ± 5
SDS	0.5	110 ± 4	123 ± 6
	1	118 ± 1	135 ± 2
	2	136 ± 6	133 ± 3
Triton X‐100	0.05% (v/v)	106 ± 1	105 ± 0
	0.1% (v/v)	107 ± 3	100 ± 2
	0.2% (v/v)	113 ± 1	101 ± 0

^a^ The TYR activity of *Hc*Tyr1 and *Hc*Tyr2 measured under standard conditions was taken as 100% (no inhibitors added), 20 mm MES pH 5.5 was used for both TYRs. Both enzymes were in latent form.

The low number of reports where the influence of metal ions on TYR activity was studied makes it difficult to uncover a trend or any fundamental principle (Table S1). Thus far, it appears that the same metal will affect TYRs differently. Furthermore, the diverse and variable effect of some metal ions on activity could be explained first by the concentration range used [45, 62, 68, 69] and second by the different surface protein residues and their interaction with some metal ions, which can cause conformational changes, protein aggregation and/or precipitation, thus hampering activity [70, 71, 72, 73, 74]. In addition to such explanations, other circumstances such as metal ion replacement in the active site can prevent protein activity, particularly Zn^2+^ ions that compete with Cu^2+^ for protein metal binding sites.

Regarding the chemical agents, SDS is an anionic surfactant widely applied in protein structural studies to break the hydrophobic interactions, ionic bonds and hydrogen bonds [75, 76, 77, 78]. It is also known to be an activating agent of TYRs [79]. This activating effect is not limited to TYRs with a LID such as we observed in *Hc*Tyr1 and *Hc*Tyr2. The activating effect was also observed in TYRs without a LID such as *Pm*Tyr from *P. megaterium* (formerly classified as *B. megaterium*) [44] with a 15–50% activity increase in 2–50 mm SDS [79], or CZA14Tyr from *Streptomyces pharetrae* (117 ± 3% relative activity at 50 mm SDS) [80].

Although NaCl is the main salinity component in seawater constituting almost 90% (w/w) of all the salts (seawater has 0.6 m NaCl), it is inhibitory to most enzymes because it limits their efficiency in biological settings [81, 82]. Indeed, NaCl is known to be a natural TYR inhibitor and is used as an anti‐browning agent in the food industry [83, 84, 85, 86]. However, in the present study, the NaCl tolerance of *Hc*Tyr1 and *Hc*Tyr2 is demonstrated, especially *Hc*Tyr1, which is stimulated by NaCl at 0.5 m (121 ± 5% relative activity) and still exhibits 90% relative activity at 5 m NaCl (Table 1). This enzymatic behaviour is not unusual for enzymes that originate rom halophilic environments where NaCl is abundant. Indeed, NaCl can be obligatory for enzymatic activity in some cases [87]. However, there are instances where NaCl is detrimental for activity, such is the case of *Hc*Tyr2 with only 58 ± 3% activity in 0.25 m NaCl. In the present study, *Hc*Tyr1 was highly resistant to chloride ions, indicating that this enzyme can be active in the varying salt concentrations, typical in costal mangrove forests.

### Kinetic parameters of *Hc*Tyr1 and *Hc*Tyr2

*Hc*Tyr1 and *Hc*Tyr2 TYRs showed typical Michaelis–Menten type kinetics determined with l‐DOPA and l‐tyrosine as substrates, with monophenolase (cresolase activity, EC 1.14.18.1) and diphenolase (catecholase activity, EC 1.10.3.1) activities, respectively. The *K*_M_, and *k*_cat_ values were determined for l‐tyrosine (*Hc*Tyr1: 0.187 ± 0.035 mm, 0.71 ± 0.03 s^−1^; *Hc*Tyr2: 0.160 ± 0.005 mm, 6.12 s^−1^) and l‐DOPA (*Hc*Tyr1: 0.370 ± 0.005 mm, 1.00 ± 0.0 s^−1^; *Hc*Tyr2: 0.196 ± 0.006 mm, 9.94 ± 0.0 s^−1^). The absence of the LID (cleaved‐*Hc*Tyr) significantly increases the affinity and turnover rate of *Hc*Tyr1 for both substrates (l‐tyrosine: 0.147 ± 0.03 mm, 21.8 ± 1 s^−1^; l‐DOPA: 0.347 ± 0.007 mm, 51.3 ± 0.5 s^−1^), indicating that the LID is hampering substrate accessibility to the active site. Comparing both TYRs in pro form, on the one hand *Hc*Ty2 exhibited higher *k*_cat_ than *Hc*Ty1 for l‐tyrosine and l‐DOPA, but, on the other hand, *Hc*Ty1 has slightly higher substrate affinity than *Hc*Tyr2. Furthermore, both TYRs have higher substrate affinity for l‐tyrosine than l‐DOPA but higher turnover rates for l‐DOPA than ‐tyrosine. All of the kinetic parameters determined here for *Hc*Tyr1 and *Hc*Tyr2 are generally similar to those reported for other bacteria, such as *Bt*Tyr (l‐tyrosine: 0.563 mm, 6.67 s^−1^; l‐DOPA: 0.768 mm, 28.3 s^−1^) [88], *Ba*Tyr (0.163 mm, 2.55 s^−1^; 0.288 mm, 3.76 s^−1^) or *Pp*Tyr (0.23 mm; 0.33 mm) [53], but instances of higher affinities can also be found; for example, in *Pm*Tyr from *P. megaterium*, formerly classified as *B. megaterium* [44] (0.075 mm, 2.5 s^−1^; 0.35 mm, 10.1 s^1^) [45], as well as cases of lower affinities reported in *V. tyrosinaticus* TYR (3.1 mm; 36 mm) [89] or *Candidatus Nitrosopumilus koreensis* (9.2 mm, 4.3 s^−1^; 2.6 mm, 2.2 s^−1^) to but name a few. Other examples can be found through the BRENDA server (http://www.brenda‐enzymes.org) [90], although the general trend is that the monophenolase activity is usually lower than diphenolase activity and *Hc*Tyr1 and *Hc*Tyr2 fall into that trend.

### Substrate profile with emphasis on lignin

Besides the substrates chosen for kinetic measurements, *Hc*Tyr1 and *Hc*Tyr2 activity was also tested against coniferyl alcohol, *p*‐coumaric acid, lignin (alkali), phenol, pyrocatechol, syringic acid, syringol, sinapic acid, pyrogallol, veratryl alcohol and coniferyl aldehyde (Table 2, Fig. S13). These substrates reflect phenolic and polyphenolic compounds that *Hc*Tyr1 and *Hc*Tyr2 may encounter in their natural environment and that are part of the ‘latch mechanism’.

**Table 2:** The substrate acceptance of *Hc*Tyr1 and *Hc*Tyr2. Enzymatic activities are given in a qualitative manner considering colour formation and the change in absorbance over time: change visible after 2–30 s (+++), after 30 s to 30 min (++), after 30 min to 16 h (+) and no discernible activity (−). ND, not detected.

Substrate	Concentration (mm)	Activity	
		*Hc*Tyr1	*Hc*Tyr2
Coniferyl alcohol	0.5	+	+
Coniferyl aldehyde	0.5	+	+
Lignin (alkali)	Unknown^a^	−	−
*p*‐Coumaric acid	0.5	+	+
Phenol	0.5	++	++
Pyrocatechol	0.5	++	++
Pyrogallol	0.5	+	+
Sinapic acid	0.5	−	−
Syringic acid	0.5	+	+
Syringol	0.5	+	+
Tyrosine	0.25	+++	+++
Veratryl alcohol	0.5	+	+
Dimethylsulfoxide^b^	2.5% (v/v)	ND	ND

^a^ Lignin was ‘dissolved’ in 20 mm MES, pH 5.5, 25% (v/v) dimethylsulfoxide, sonicated and centrifuged. The supernatant was used as substrate stock.

^b^ Solvent negative control.

Activity on *p*‐coumaric acid, phenol, pyrocatechol, syringol and pyrogallol could be observed by the naked eye for *Hc*Tyr1 and *Hc*Tyr2 as a result of the formation of coloured products (Fig. S14). However, no observable colour could be detected on the remaining substrates (coniferyl alcohol, coniferyl aldehyde, lignin, syringic acid, sinapic acid and veratyrl alcohol); therefore, activity was evaluated by recording the absorbance spectrum (wavelength 230–600 nm) for each reaction and negative control to detect product formation or substrate depletion over time. The absorbance spectrum recording over time revealed a clear activity of *Hc*Tyr1 and *Hc*Tyr2 on coniferyl alcohol, coniferyl aldehyde, syringic acid, sinapic acid and veratyrl alcohol (Figs S15 and S16). However, these activities are only marginal compared to the native substrates and, in some cases, these substrates can even be inactivating the enzyme over time. When lignin was used as substrate, there was no clear difference between the reaction with substrate and the negative control where a similar decrease rate for both was observed (Fig. S17). Despite not showing a clear activity on lignin, because it can in fact be a TYR inhibitor [91], *Hc*Tyr1 and *Hc*Tyr2 are able to accept several of the constituents of lignin tested here.

### Crystallization and model analysis

*Hc*Tyr1 and *Hc*Tyr2 crystallized in the conditions described in 2.10. *Hc*Tyr1 formed crystals containing the full‐length sequence in orthorhombic space group P 2 2 2_1_ originating a unit cell of *a* = 51.11 Å, *b* = 55.74 Å, *c* = 155.23 Å, α = β = γ = 90.00° that diffracted to a resolution of 1.9 Å (for further statistics, see Table S2). *Hc*Tyr2 crystals, however, diffracted to a low resolution of 7–8 Å, rendering structural elucidation not possible (data collected by the author GAS). We are currently attempting to soak *Hc*Tyr1 holo‐form crystals in copper and crystallize *Hc*Tyr2 under different conditions; for this reason, we used the *Hc*Tyr2 Alphafold2 (AF2) model for comparison.

**Fig. 11:**
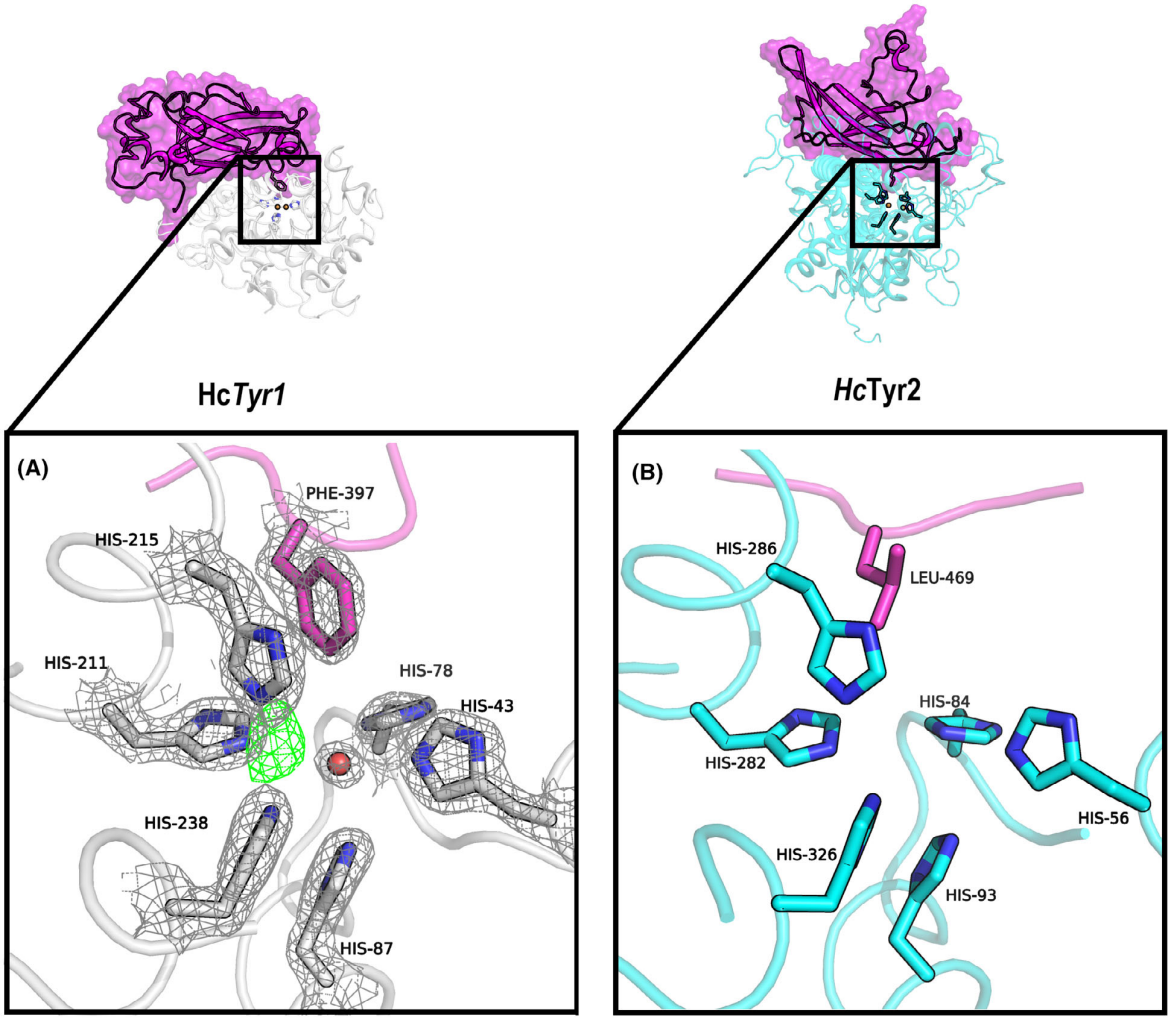
The active site comparison between the crystal structure of (A) *Hc*Tyr1 and (B) the Alphafold model of *Hc*Tyr2. Active site residues are shown as sticks; oxygen from a water molecule is represented as a sphere, coloured in red. Placeholder residues Phe397 (*Hc*Tyr1) and Leu469 (*Hc*Tyr2) are coloured magenta. 2*F*_o_ − *F*_c_ electron‐density mesh (2σ), grey; *F*_o_ − *F*_c_ electron‐density mesh (3σ), green. LID‐domains are coloured in magenta; *Hc*Tyr1 TYR‐domain are coloured in white; and *Hc*Tyr2 TYR‐Domain are coloured in cyan.

Both TYRs have their LID with different placeholder residues to the TYR active site: a phenylalanine (Phe397) in *Hc*Tyr1 and a leucine (Leu469) in *Hc*Tyr2 ( Fig. 11). Interestingly, the majority of identified TYRs have a tyrosine or a phenylalanine at the corresponding position even though other residues can exist at the corresponding position: valine, glycine, alanine, glutamic acid, asparagine or even threonine [92]. However, the placeholder residue leucine is less common but may have similar function as tyrosine or phenylalanine given its similar hydrophobicity and size (Fig. S18). Interestingly, the presence of a leucine (Leu469) in *Hc*Tyr2 (instead of a bulkier aromatic residue such as Phe397 in *Hc*Tyr1) may very well be decisive for *Hc*Tyr2 being active even in the presence of the LID‐domain, whereas *Hc*Tyr1 (having a Phe) benefits from the LID removal, having increased activity levels, affinity for substrate and turnover rate. *Hc*Tyr1 is the second prokaryotic TYR in the PDB with a LID domain. There are structures of two TYRs, from *S. castaneoglobisporus* [93] and *Streptomyces avermitilis* (unpublished data) that have caddy proteins associated; however, these rather small proteins are translated as independent entities. The TYR *Bt*TYR from *Bu. thailandensis* [47] has a LID domain of similar size to the one found in *Hc*Tyr1, but the PDBeFOLD [94] 3D alignment of the structures, returning a rmsd value of 2.7 Å and a *Q* value of 0.214, indicates significant differences. Indeed, the LID domain and overall structure overlay poorly upon visual inspection. In *Hc*Tyr1, Phe397 is protruding towards the active site where electron density for an oxygen atom from a water molecule was observed (instead of two copper ions, CuA and CuB) along with six histidines (His43, His78, His87, His211, His215 and His238) (Fig. 11). His43, His78 and His87 will coordinate CuA, and His211, His215 and His238 will coordinate CuB. The same six‐histine configuration is modelled in *Hc*Tyr2 by AF2 where His56, His84 and His93 will coordinate CuA, and His282, His286 and His326 will coordinate CuB. In *Hc*Tyr1, a positive electron density (*F*_o_ − *F*_c_) near His211, His215 and His238 was detected, likely caused by another water molecule. Furthermore, despite Cys76 displaying two alternative configurations (occupancy 0.53 and 0.47), no thioether bridge was found between Cys76 and His78 or His43; however, several hydrogen bonds possibilities are present with His78 (Fig. S19).

The AF2 model of *Hc*Tyr1 aligned with our crystal structure with a rmsd of 0.82 Å, which makes them almost exact copies of each other. Furthermore, for the areas where no electron density was detected in our crystal structure, the linker between the TYR and LID (residues 302–314) and two loops (residues 338–356 and 407–408) are highly flexible regions that, in the AF2 model, exhibit lower per‐residue confidence score (Fig. S20).

## Conclusions

In summary, the two TYRs from *Hahella* sp. CCB MM4 (*Hc*Tyr1 and *Hc*Tyr2) have been cloned, expressed, purified and characterized biochemically and biophysically. The crystal structure of *Hc*Tyr1 has been elucidated, and it displays structural differences compared to other TYRs in the PDB and represents a cluster of nodes in our sequence similarity network of bacterial TYRs that has not previously been characterized. Our data clearly demonstrate the sparsity of the experimental data for bacterial TYRs. *Hc*Tyr1 and *Hc*Tyr2 exhibited catalytic activity in the psychrophile to a mesophile temperature range with distinctive differences in pH optima and applicable pH ranges. *Hc*Tyr1 exhibited high saline tolerance, which may reflect a specialized function in the varying environment of the host habitat. The crystal structure of *Hc*Tyr1 reveals a LID domain that is different from other characterized bacterial TYRs, and our data indicate that the LID domain is important for both activity and stability. Furthermore, the LID domain of *Hc*Tyr1 was cleaved off in an autoprocessive time dependent manner, and the processing rate was not influenced by factors such as pH and added metal ions. Finally, the ability of *Hc*Tyr1 and *Hc*Tyr2 to accept several phenolic compounds as substrate was demonstrated to potentially participate in lignin functionalization.

## Conflicts of interest

The authors declare that they have no conflicts of interest.

### Peer review

The peer review history for this article is available at https://www.webofscience.com/api/gateway/wos/peer‐review/10.1002/2211‐5463.13906.

## Author contributions

GAS was responsible for conceptualization. GAS was responsible for experimental work, analysis, writing of manuscript, review and editing. GAS, ANBE, ELD and ÅRK were responsible for analysis. GAS, ELD and ÅRK were responsible for writing the manuscript. GAS was responsible for reviewing and editing. ANBE was responsible for structural elucidation review. ELD was responsible for bioinformatics and graphical abstract preparation. ÅRK was responsible for resources, review, supervision, project administration and funding acquisition.

## Supporting information


**Fig. S1.** Any kD™ Mini‐PROTEAN TGX Stain‐Free™ polyacrylamide gel analysis of the different fractions obtained during purification of (A) *Hc*Tyr1 approximately 52 kDa and (B) *Hc*Tyr2 approximately 61 kDa.
**Fig. S2.** Any kD™ Mini‐PROTEAN TGX Stain‐Free™ polyacrylamide gel analysis of purified *Hc*Tyr1 stored at 4 °C.
**Fig. S3.** Any kD™ Mini‐PROTEAN TGX Stain‐Free™ polyacrylamide gel analysis of purified *Hc*Tyr1 stored for at 4 °C to investigate the effect of pH in inducing the cleavage of the lid over a 1‐week period.
**Fig. S4.** Any kD™ Mini‐PROTEAN TGX Stain‐Free™ polyacrylamide gel analysis of purified *Hc*Tyr1to investigate the effect of transition metal ion mix on the cleavage of the lid over a 24‐h period.
**Fig. S5.** Any kD™ Mini‐PROTEAN TGX Stain‐Free™ polyacrylamide gel analysis of purified *Hc*Tyr1to investigate the effect of pH and transition metal ion mix on the cleavage of the lid.
**Fig. S6.** Any kD™ Mini‐PROTEAN TGX Stain‐Free™ polyacrylamide gel analysis of purified *Hc*Tyr1 after a 16‐h incubation at 37 °C with and without protease inhibitors (EDTA 10 mm and 1 × cOmplete EDTA‐free) (Sigma‐Aldrich).
**Fig. S7.** The surface charge of the LID and TYR of *Hc*Tyr1 and *Hc*Tyr2 at pH 5 and pH 8.
**Fig. S8.** Any kD™ Mini‐PROTEAN TGX Stain‐Free™ polyacrylamide gel analysis of the different pH incubations at 4 °C in the absence (A) and presence (B) of 140 μMm CuCl_2_.
**Fig. S9.** Any kD™ Mini‐PROTEAN TGX Stain‐Free™ polyacrylamide gel analysis of the different pH incubations at 4 °C in the presence of 140 μm CuCl_2_.
**Fig. S10.** Any kD™ Mini‐PROTEAN TGX Stain‐Free™ polyacrylamide gel analysis of the incubations at pH 2 and pH 3 and 4 °C in the presence of 140 μMm CuCl_2_.
**Fig. S11.** The *Hc*Tyr1 Alphafold2 model cartoon representation.
**Fig. S12.** Any kD™ Mini‐PROTEAN TGX Stain‐Free™ polyacrylamide gel analysis of the temperature influence on latent‐*Hc*Tyr1 dimer formation at pH 7.
**Fig. S13.** The chemical structures of the tested substrates.
**Fig. S14.** The substrate acceptance test for cleaved‐*Hc*Tyr1 and *Hc*Tyr2.
**Fig. S15.** Recorded absorbance spectrum of the reaction of *Hc*Tyr1 and the respective negative controls over time.
**Fig. S16.** Recorded absorbance spectrum of the reaction of *Hc*Tyr2 and the respective negative controls over time.
**Fig. S17.** Recorded absorbance spectrum of the reaction of *Hc*Tyr1 (left) and *Hc*Tyr2 (right) with lignin and the respective negative controls over time.
**Fig. S18.** Placeholder residue of *Hc*Tyr1, *Hc*Tyr2 (both AF2 models) and *Sc*Tyr (PDB: 1WX2).
**Fig. S19.** The two Cys76 alternative conformations in the *Hc*Tyr1 active site.
**Fig. S20.** Side‐by‐side cartoon representation of *Hc*Tyr1 crystal structure (a) and its alphafold2 model (b).
**Table S1.** The relative activities of tyrosinases from different organisms in the presence of different metals at 1 mm.
**Table S2.** The data collection and refinement statistics for recombinant *Hc*Tyr1.

## Data Availability

The protein sequence data that support the findings of the present study are openly available in UniProt (https://www.uniprot.org), primary accession numbers IPR008922, A0A261GRE4 and A0A261GVB1. The structural data that support these findings are openly available in the wwPDB at https://www.wwpdb.org/pdb?id=pdb_00008b74.
