# Supplementary material for: Characterization of two bacterial tyrosinases from the halophilic bacterium Hahella sp. CCB MM4 relevant for phenolic compounds oxidation in wetlands
Source: FEBS Open Bio. 2024 Oct 9;14(12):2038–58. doi: 10.1002/2211-5463.13906 (PMC11609597; doi:10.1002/2211-5463.13906)
Supplement: Supplementary file 1 — Fig. S1. Any kD™ Mini‐PROTEAN TGX Stain‐Free™ polyacrylamide gel analysis of the different fractions obtained during purification of (A) HcTyr1 approximately 52 kDa and (B) HcTyr2 approximately 61 kDa. Fig. S2. Any kD™ Mini‐PROTEAN TGX Stain‐Free™ polyacrylamide gel analysis of purified HcTyr1 stored at 4 °C. Fig. S3. Any kD™ Mini‐PROTEAN TGX Stain‐Free™ polyacrylamide gel analysis of purified HcTyr1 stored for at 4 °C to investigate the effect of pH in inducing the cleavage of the lid over a 1‐week period. Fig. S4. Any kD™ Mini‐PROTEAN TGX Stain‐Free™ polyacrylamide gel analysis of purified HcTyr1to investigate the effect of transition metal ion mix on the cleavage of the lid over a 24‐h period. Fig. S5. Any kD™ Mini‐PROTEAN TGX Stain‐Free™ polyacrylamide gel analysis of purified HcTyr1to investigate the effect of pH and transition metal ion mix on the cleavage of the lid. Fig. S6. Any kD™ Mini‐PROTEAN TGX Stain‐Free™ polyacrylamide gel analysis of purified HcTyr1 after a 16‐h incubation at 37 °C with and without protease inhibitors (EDTA 10 mm and 1 × cOmplete EDTA‐free) (Sigma‐Aldrich). Fig. S7. The surface charge of the LID and TYR of HcTyr1 and HcTyr2 at pH 5 and pH 8. Fig. S8. Any kD™ Mini‐PROTEAN TGX Stain‐Free™ polyacrylamide gel analysis of the different pH incubations at 4 °C in the absence (A) and presence (B) of 140 μMm CuCl2. Fig. S9. Any kD™ Mini‐PROTEAN TGX Stain‐Free™ polyacrylamide gel analysis of the different pH incubations at 4 °C in the presence of 140 μm CuCl2. Fig. S10. Any kD™ Mini‐PROTEAN TGX Stain‐Free™ polyacrylamide gel analysis of the incubations at pH 2 and pH 3 and 4 °C in the presence of 140 μMm CuCl2. Fig. S11. The HcTyr1 Alphafold2 model cartoon representation. Fig. S12. Any kD™ Mini‐PROTEAN TGX Stain‐Free™ polyacrylamide gel analysis of the temperature influence on latent‐HcTyr1 dimer formation at pH 7. Fig. S13. The chemical structures of the tested substrates. Fig. S14. The substrate acceptance test for cleaved‐HcTyr1 and HcTyr2. [file FEB4-14-2038-s001.docx]

## Supporting Information

## Figures

| 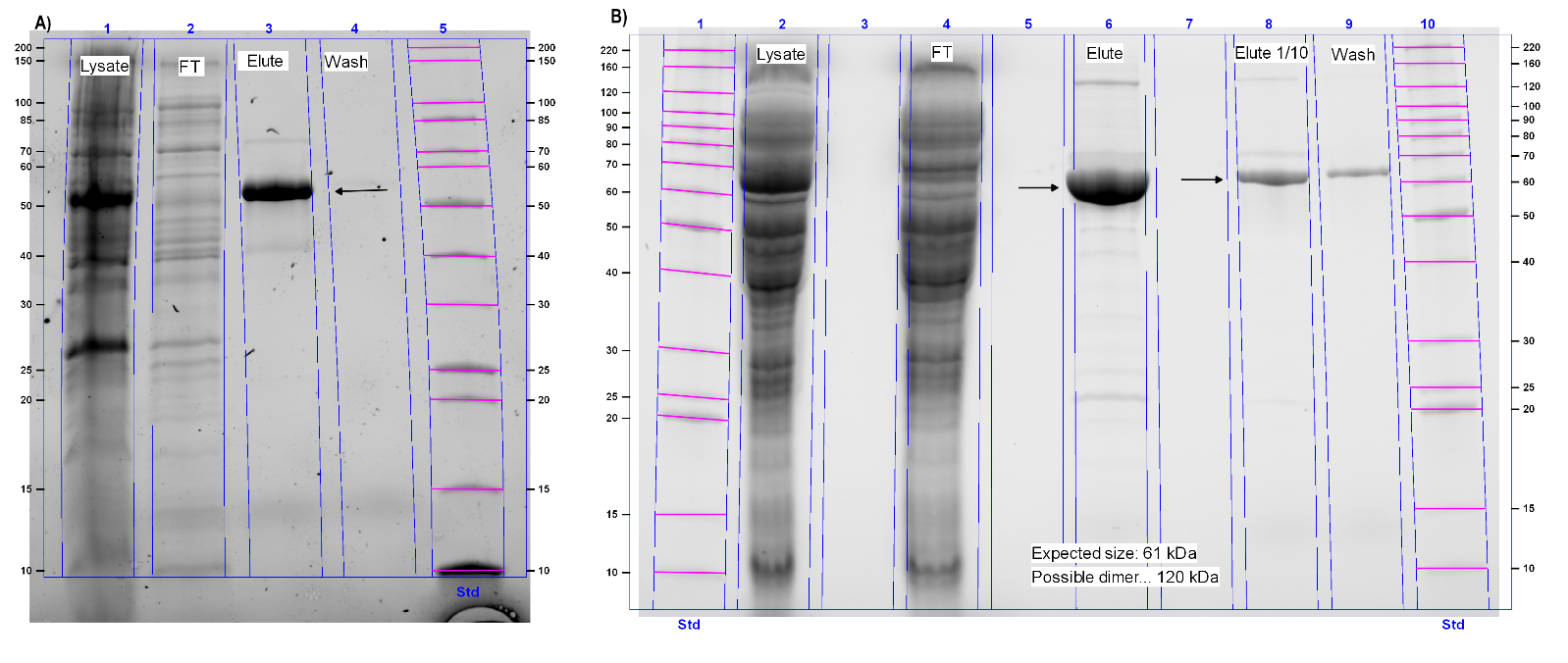 |
| --- |
| **Fig. S1 –** The Any kD™ Mini-PROTEAN TGX Stain-Free™ polyacrylamide gel analysis of the different fractions obtained during purification of (A) *Hc*Tyr1 ca. 52 kDa and (B) *Hc*Tyr2 ca. 61 kDa. Arrows indicate the purified protein, FT (Flowthrough), Std (Molecular weight marker). |
| 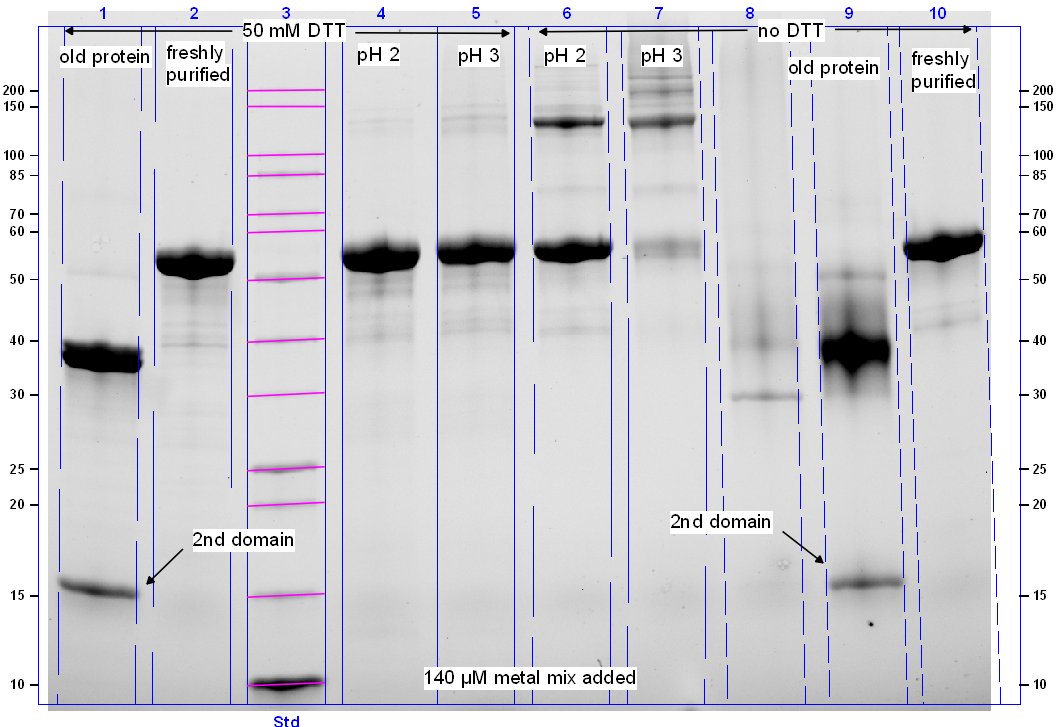 |
| **Fig. S2 –** The Any kD™ Mini-PROTEAN TGX Stain-Free™ polyacrylamide gel analysis of purified HcTyr1 stored at 4 °C. The freshly purified HcTyr1 displays the same kDa as calculated while *Hc*Tyr1 that is over 8 weeks old (stored at 4 °C) exhibits a size that coincides with the loss of the lid (highlighted by arrow). In Lanes 4 to 7 a comparison of the presence of DTT at pH 2 and pH 3 when 140 µM metal mix is added. Under reducing conditions no dimerization is observed. Copper saturated *Hc*Tyr1 concentration at 70 µM, “Universal” buffer for the different pHs: 5 mM sodium citrate, 5 mM potassium phosphate, 5 mM Tris HCl, 5 mM glycine NaOH and 5 mM Na_3_PO_4_-NaOH, Std (molecular weight marker). |
| **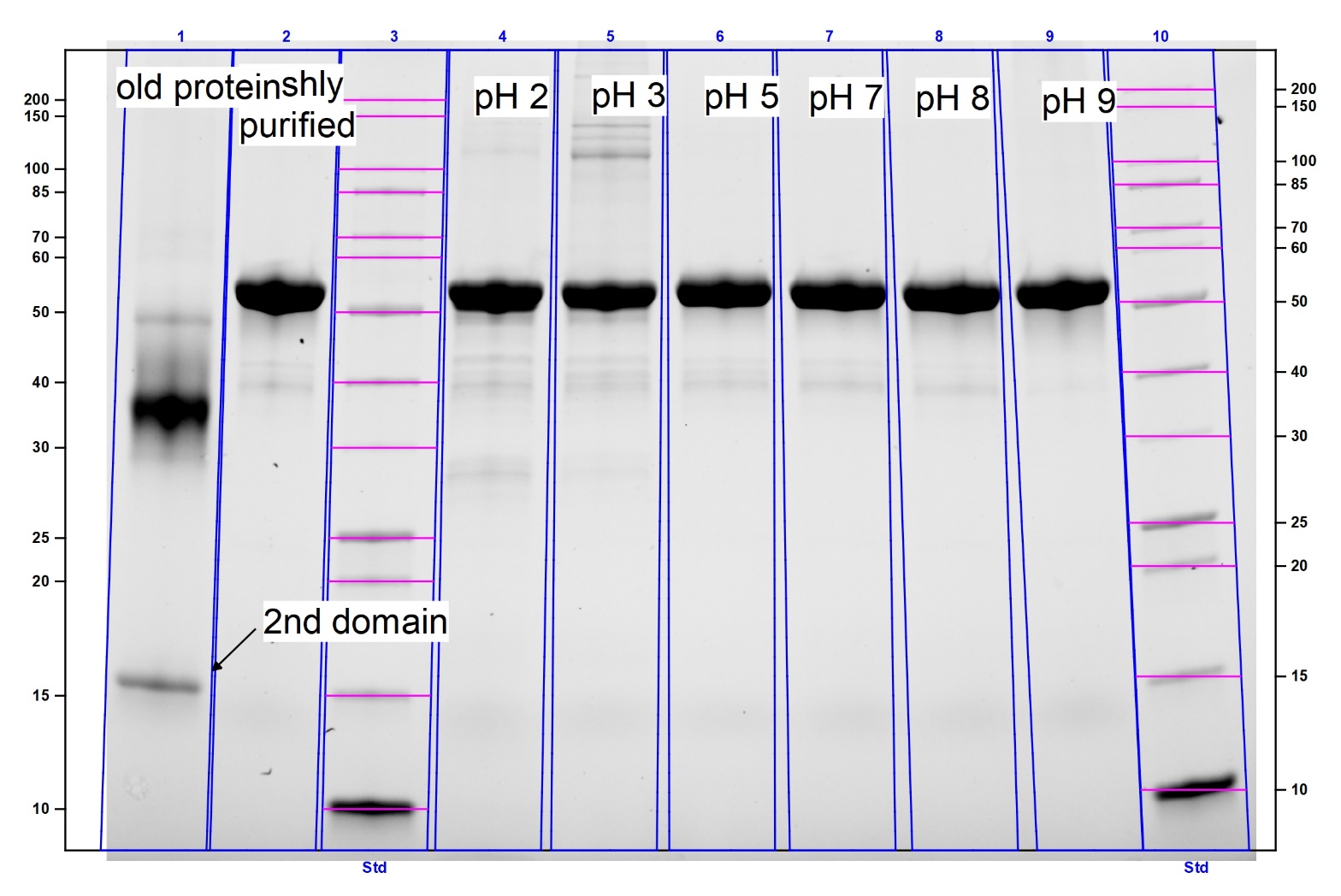** |
| **Fig. S3 –** The Any kD™ Mini-PROTEAN TGX Stain-Free™ polyacrylamide gel analysis of purified *Hc*Tyr1 stored for at 4 °C to investigate the effect of pH in inducing the cleavage of the lid over a 1-week period. The freshly purified *Hc*Tyr1 displays the same kDa as calculated while *Hc*Tyr1 that is over 8 weeks old (stored at 4 °C) exhibits a size that coincides with the loss of the lid (highlighted by arrow). The incubation at different pHs (4 to 9) did not significantly induce the cleavage of the lid over a period of 1 week at 4 °C. Copper saturated *Hc*Tyr1 concentration at 70 µM, “Universal” buffer for the different pHs: 5 mM sodium citrate, 5 mM potassium phosphate, 5 mM Tris HCl, 5 mM glycine NaOH and 5 mM Na_3_PO_4_-NaOH, Std (molecular weight marker). |
| **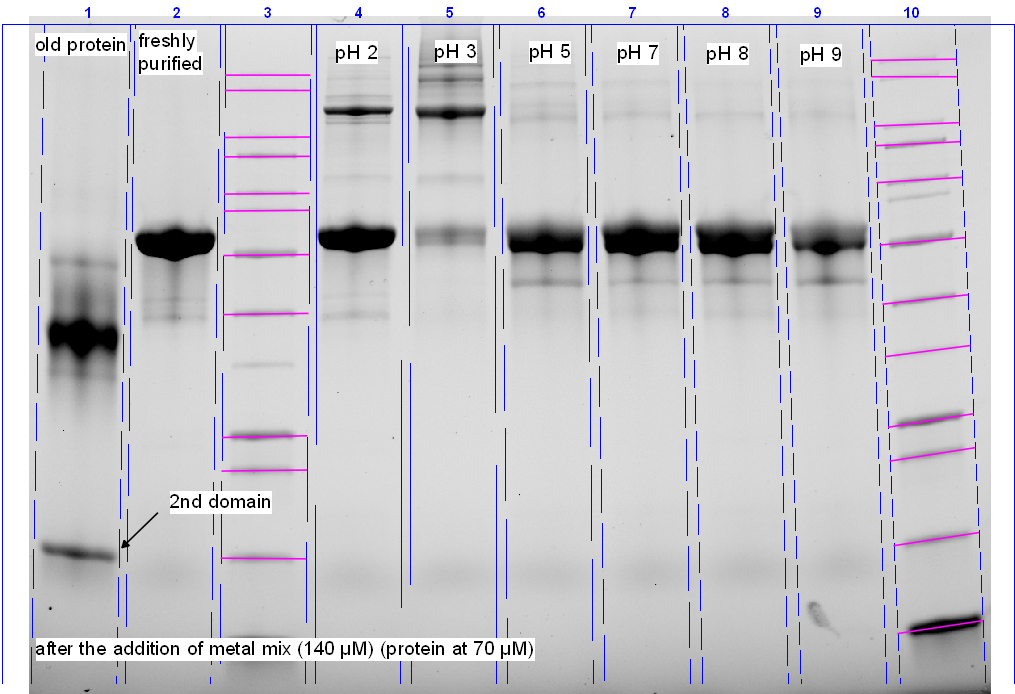** |
| **Fig. S4 –** The Any kD™ Mini-PROTEAN TGX Stain-Free™ polyacrylamide gel analysis of purified *Hc*Tyr1to investigate the effect of transition metal ion mix on the cleavage of the lid over a 24-hour period. The freshly purified HcTyr1 displays the same kDa as calculated while HcTyr1 that is over 8 weeks old (stored at 4 °C) exhibits a size that coincides with the loss of the lid (highlighted by arrow). Incubation at different pHs (4-9) with 140 µM of a transition metal ion mix (Co^2+,^ Cu^2+^, Fe^3+^, Mn^2+^, Ni^2+^ and Zn^2+^) did not significantly induce the cleavage of the lid over the tested period. Copper saturated *Hc*Tyr1 concentration at 70 µM, “Universal” buffer for the different pHs: 5 mM sodium citrate, 5 mM potassium phosphate, 5 mM Tris HCl, 5 mM glycine NaOH and 5 mM Na_3_PO_4_-NaOH, Std (molecular weight marker). |
| **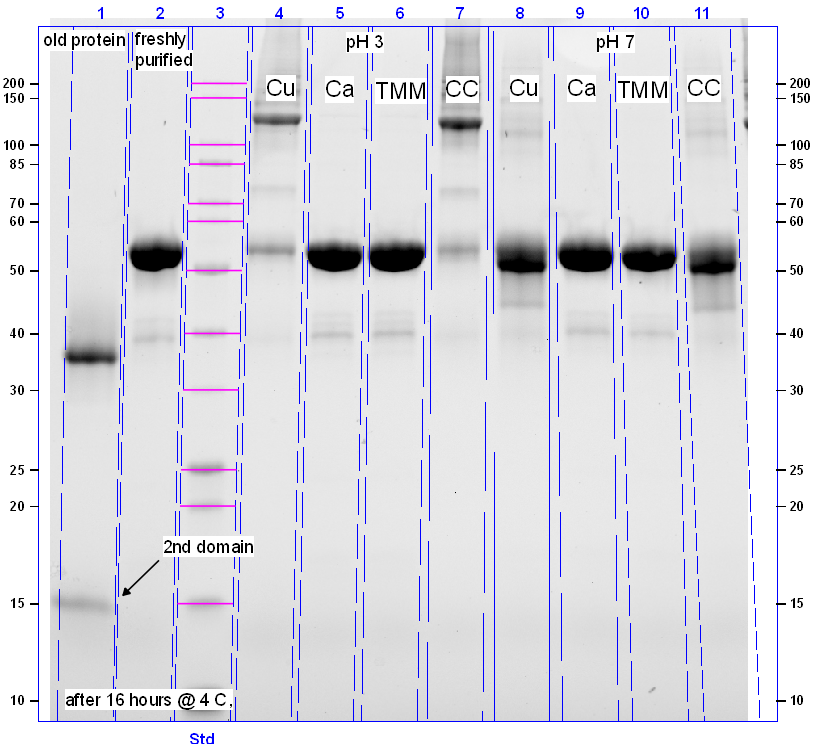** |
| **Fig. S5** – The Any kD™ Mini-PROTEAN TGX Stain-Free™ polyacrylamide gel analysis of purified *Hc*Tyr1to investigate the effect of pH and transition metal ion mix on the cleavage of the lid. The freshly purified HcTyr1 displays the same kDa as calculated while HcTyr1 that is over 8 weeks old (stored at 4 °C) exhibits a size that coincides with the loss of the lid (highlighted by arrow). Incubation at pH 3 and pH 7 with 200 µM of a CuCl_2_ (lane 4 and 8), 200 µM of CaCl_2_ (lane 5 and 9), 200 µM of a TMM (transition metal ion mix) in their chlorinated form (Co^2+^, Fe^3+^, Mn^2+^, Ni^2+^ and Zn^2+^) (lane 6 and 10) and 200 µM of CuCl_2_ and CaCl_2_ (lane 7 and 11) did not significantly induce the cleavage of the lid over a period of 16 hours at 4 °C. Copper saturated *Hc*Tyr1 concentration at 100 µM, “Universal” buffer for the different pHs: 5 mM sodium citrate, 5 mM potassium phosphate, 5 mM Tris HCl, 5 mM glycine NaOH and 5 mM Na_3_PO_4_-NaOH, Std (molecular weight marker). Cu (200 µM CuCl_2_); Ca (200 µM CaCl_2_); TMM (Transition Metal ions Mix, 200 µM Co^2+^, Fe^3+^, Mn^2+^, Ni^2+^ and Zn^2+^); CC (200 µM CuCl_2_ and CaCl_2_), Std (Molecular weight marker). |
| **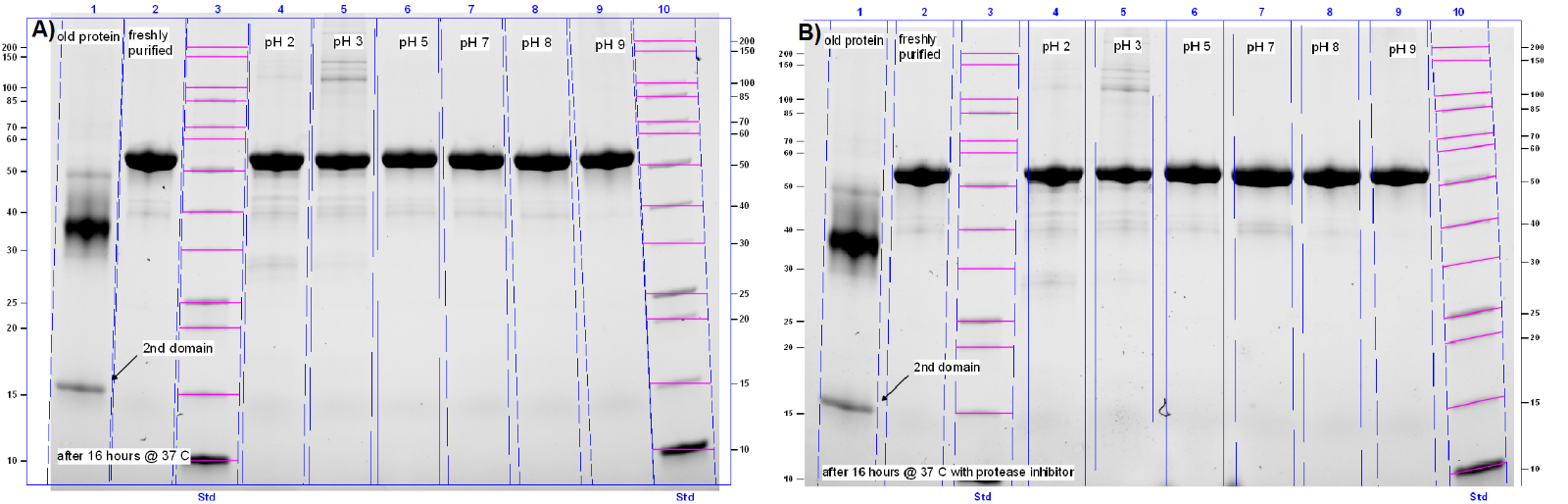** |
| **Fig. S6** – The Any kD™ Mini-PROTEAN TGX Stain-Free™ polyacrylamide gel analysis of purified *Hc*Tyr1 after a 16-hour incubation at 37 °C with and without protease inhibitors (EDTA 10 mM and 1X cOmplete EDTA-free (Sigma-Aldrich)). The freshly purified *Hc*Tyr1 displays the same kDa as calculated while *Hc*Tyr1 that is over 8 weeks old (stored at 4 °C) exhibits a size that coincides with the loss of the LID (highlighted by arrow). Incubation at the different pHs (4 to 9) at 37 °C with and without protease inhibitors did not significantly induce the cleavage of the lid over the tested period. Copper saturated *Hc*Tyr1 concentration at 70 µM, “Universal” buffer for the different pHs: 5 mM sodium citrate, 5 mM potassium phosphate, 5 mM Tris HCl, 5 mM glycine NaOH and 5 mM Na_3_PO_4_-NaOH, Std (molecular weight marker). |
| **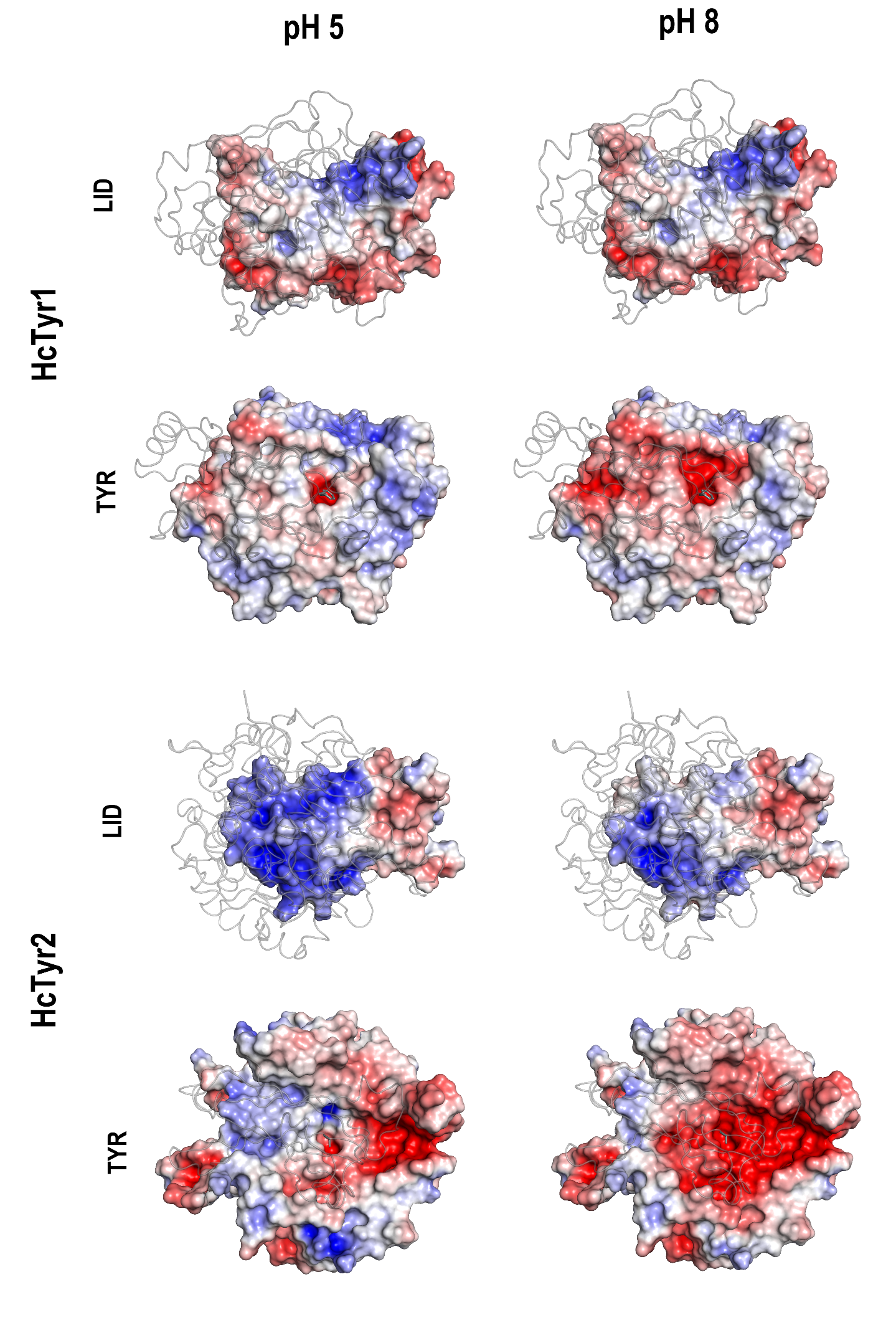** |
| **Fig. S7** – The surface charge of the LID and TYR of *Hc*Tyr1 and *Hc*Tyr2 at pH 5 and pH 8. Blue and red colours represent positive and negative charges, respectively. The figures were prepared using the output files from APBS-PDB2PQR software suite in PyMOL [94]. |
| **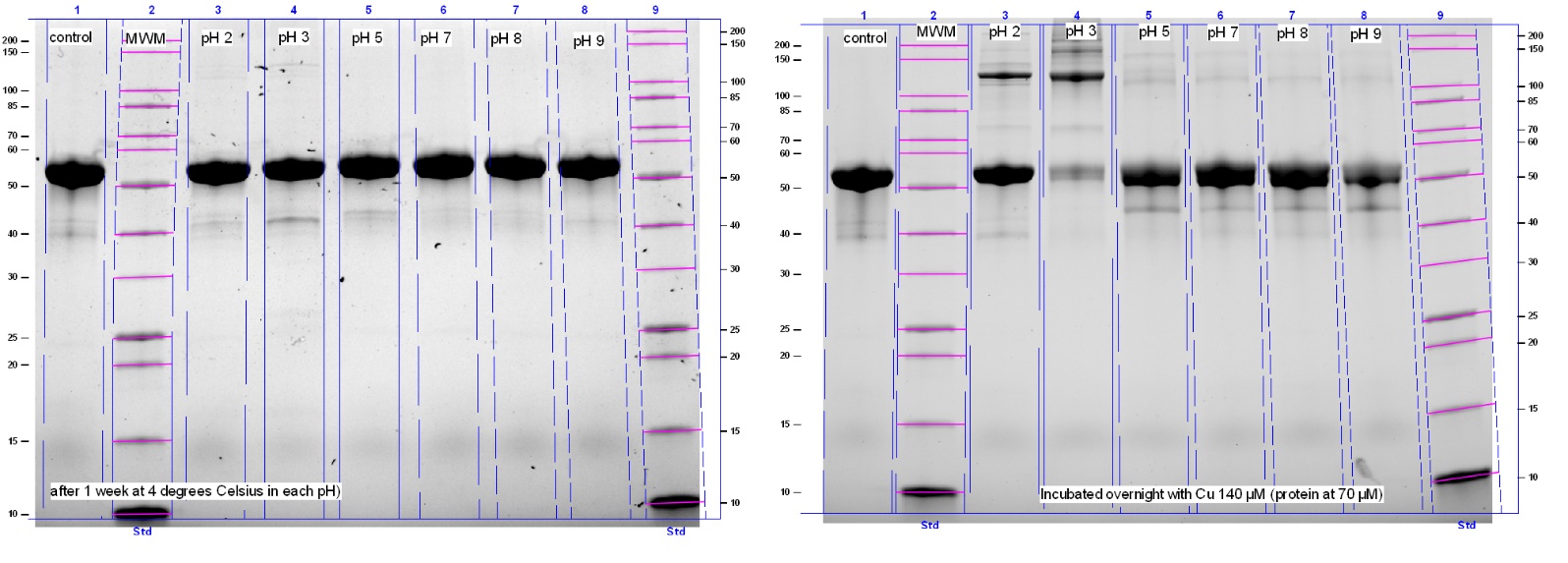** |
| **Fig. S8** – The Any kD™ Mini-PROTEAN TGX Stain-Free™ polyacrylamide gel analysis of the different pH incubations at 4 °C in the absence (A) and presence (B) of 140 µM CuCl_2_. In (B) a second band between 150 and 100 kDa can be observed at pH 2 and pH 3 (lanes 3 and 4). MWM/Std (Molecular Weight Marker), Control (protein not subjected to any treatment), Copper saturated *Hc*Tyr1 concentration at 70 µM, “Universal” buffer for the different pHs: 5 mM sodium citrate, 5 mM potassium phosphate, 5 mM Tris HCl, 5 mM glycine NaOH and 5 mM Na_3_PO_4_-NaOH. |
| **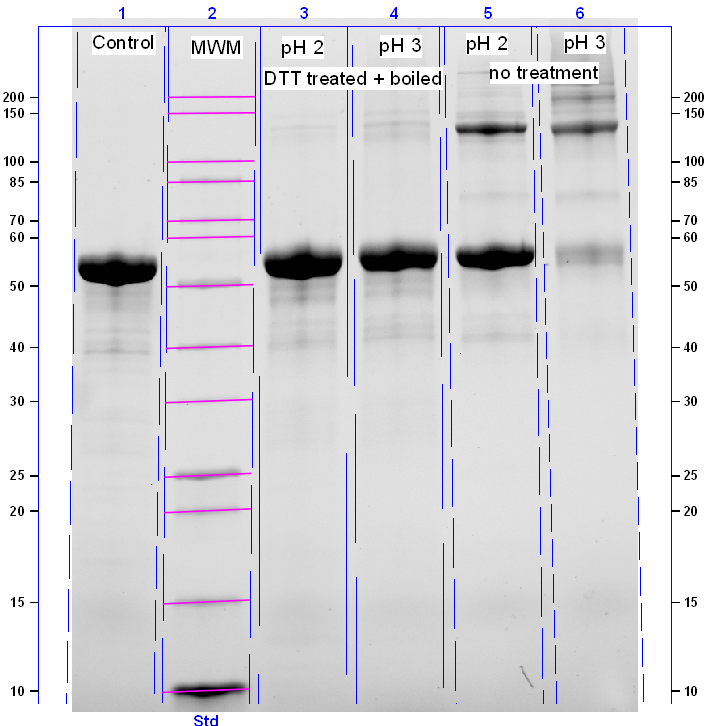** |
| **Fig. S9 –** The Any kD™ Mini-PROTEAN TGX Stain-Free™ polyacrylamide gel analysis of the different pH incubations at 4 °C in the presence of 140 µM CuCl_2_. The band observed in lane 5 and 6 between 150 and 100 kDa is no longer present once the sample is boiled (95 °C, 15 min) in reducing conditions (50 mM DTT) revealing the nature of the arrangement (covalent bond between two sulphur atoms formed by the coupling of two thiol groups). Lanes 3 and 4 the samples were treated with 50 mM DTT and heat treated at 95 °C for 15 min before being loaded on the gel. Lanes 1, 5 and 6 the samples are non-reducing conditions and were not subjected to any heat treatment. MWM/Std (Molecular Weight Marker), Control (protein not subjected to any treatment), Copper saturated *Hc*Tyr1 concentration at 70 µM, “Universal” buffer for the different pHs: 5 mM sodium citrate, 5 mM potassium phosphate, 5 mM Tris HCl, 5 mM glycine NaOH and 5 mM Na_3_PO_4_-NaOH. |
| **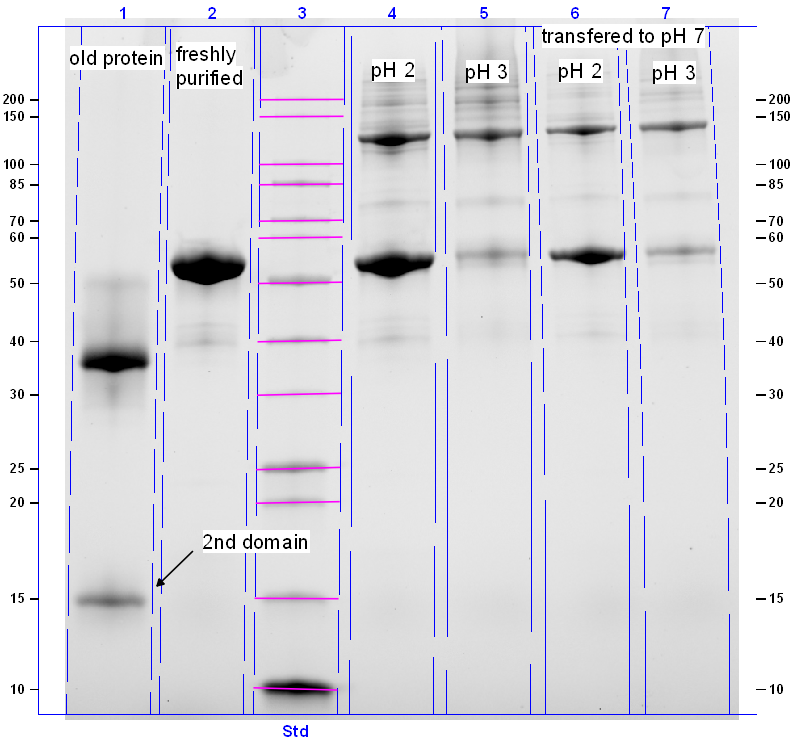** |
| **Fig. S10** **–** The Any kD™ Mini-PROTEAN TGX Stain-Free™ polyacrylamide gel analysis of the pH 2 and pH 3 incubations at 4 °C in the presence of 140 µM CuCl_2_. The band observed in lane 4 and 5 between 150 and 100 kDa is still present after a transfer to pH 7 (lane 6 and 7). lid domain is highlighted by arrow. Copper saturated HcTyr1 concentration at 70 µM, “Universal” buffer for the different pHs: 5 mM sodium citrate, 5 mM potassium phosphate, 5 mM Tris HCl, 5 mM glycine NaOH and 5 mM Na_3_PO_4_-NaOH, Std (Molecular Weight Marker). |
| **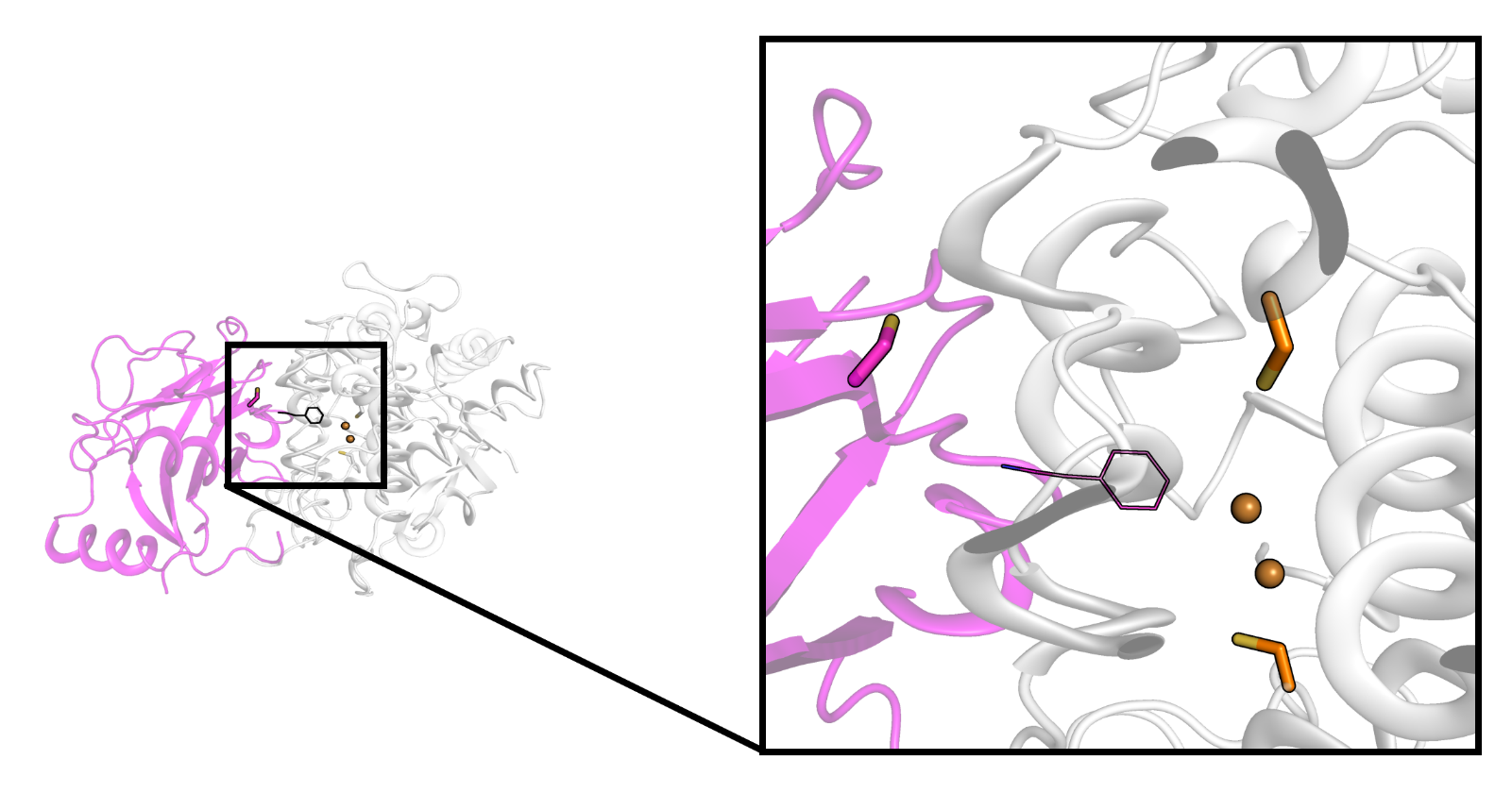** |
| **Fig. S11** – The *Hc*Tyr1 Alphafold2 model cartoon representation. Cysteines are represented as sticks, placeholder residue Phe397 as lines, copper atoms in the active site (positioned using the crystal structure with PDBid 3NM8 as template) are shown as orange spheres. The image was prepared with PyMOL v.2.4.1. |
| **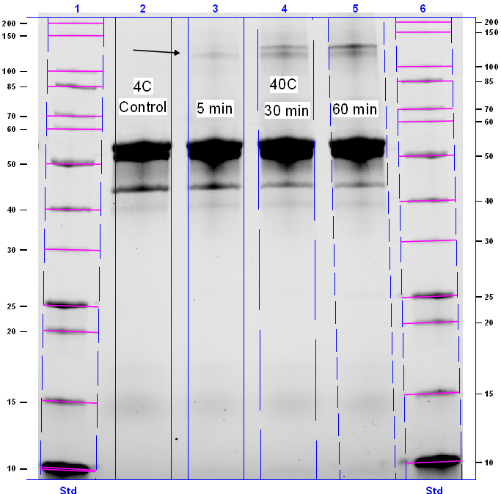** |
| **Fig. S12 –** The Any kD™ Mini-PROTEAN TGX Stain-Free™ polyacrylamide gel analysis of the temperature influence on latent-*Hc*Tyr1 dimer formation at pH 7. Two bands between 150 and 100 kDa, increasing in intensity as the incubation at 40 °C proceeds (Lane 3 ,4 and 5) are observed. Arrow highlights the formation of the dimer. Std (Molecular Weight Marker). |
| **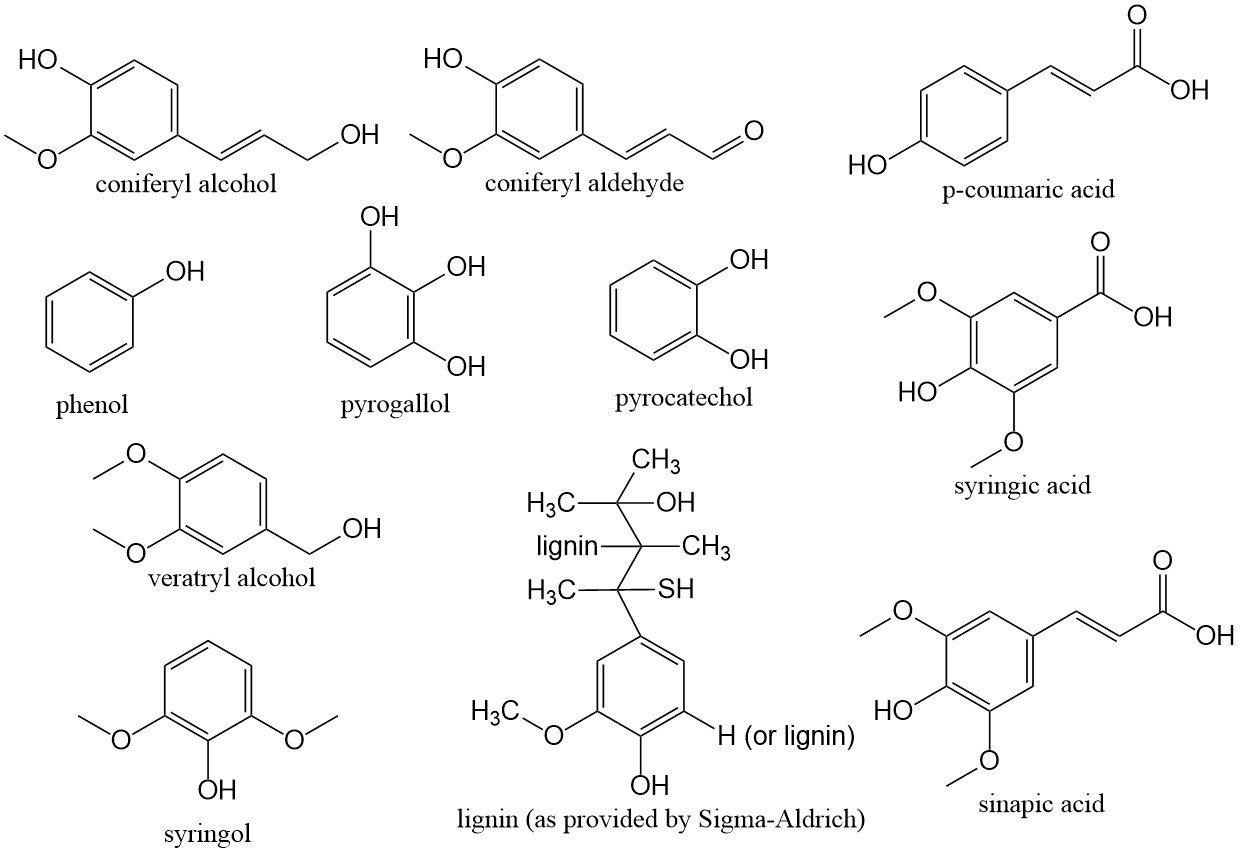** |
| **Fig. S13** – The structures of the tested substrates. Figure was prepared using ChemDraw v.19. |
| **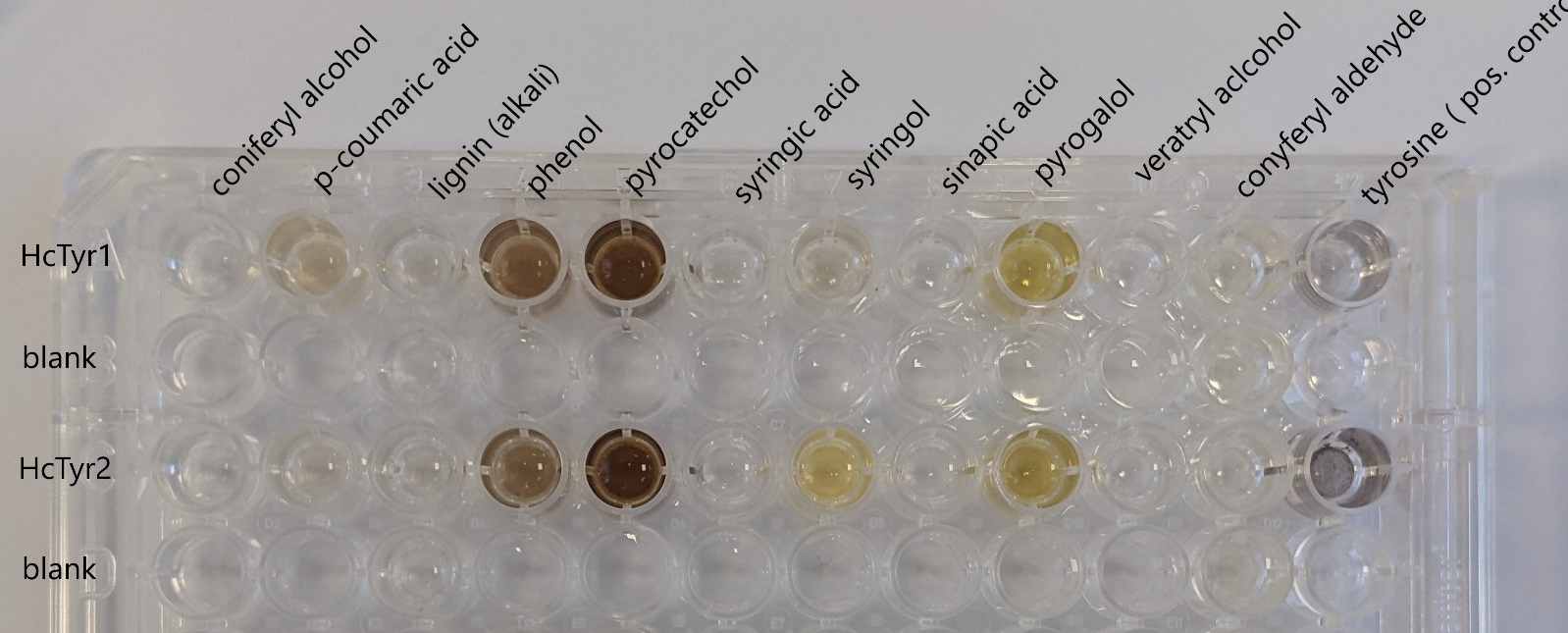** |
| **Fig. S14** – The substrate acceptance test for cleaved‑*Hc*Tyr1 and *Hc*Tyr2. The reaction solutions in row A and C contain *Hc*Tyr1 and *Hc*Tyr2 respectively while row B and D the substrate solutions were incubated without addition of enzyme (negative control). Enzymatic reactions were carried out in a total volume of 250 µL containing 20 mM MES pH 5.5, 20 µL *Hc*Tyr1 (0.05 mg/mL) or *Hc*Tyr2 (0.3 mg/mL) (or the equivalent volume of water in the negative control row) and 25 µL substrate (at 5 mM, dissolved in 20 mM MES pH 5.5, 25 % (*v/v*) DMSO). The picture was taken after 20 hours of reaction. |
| **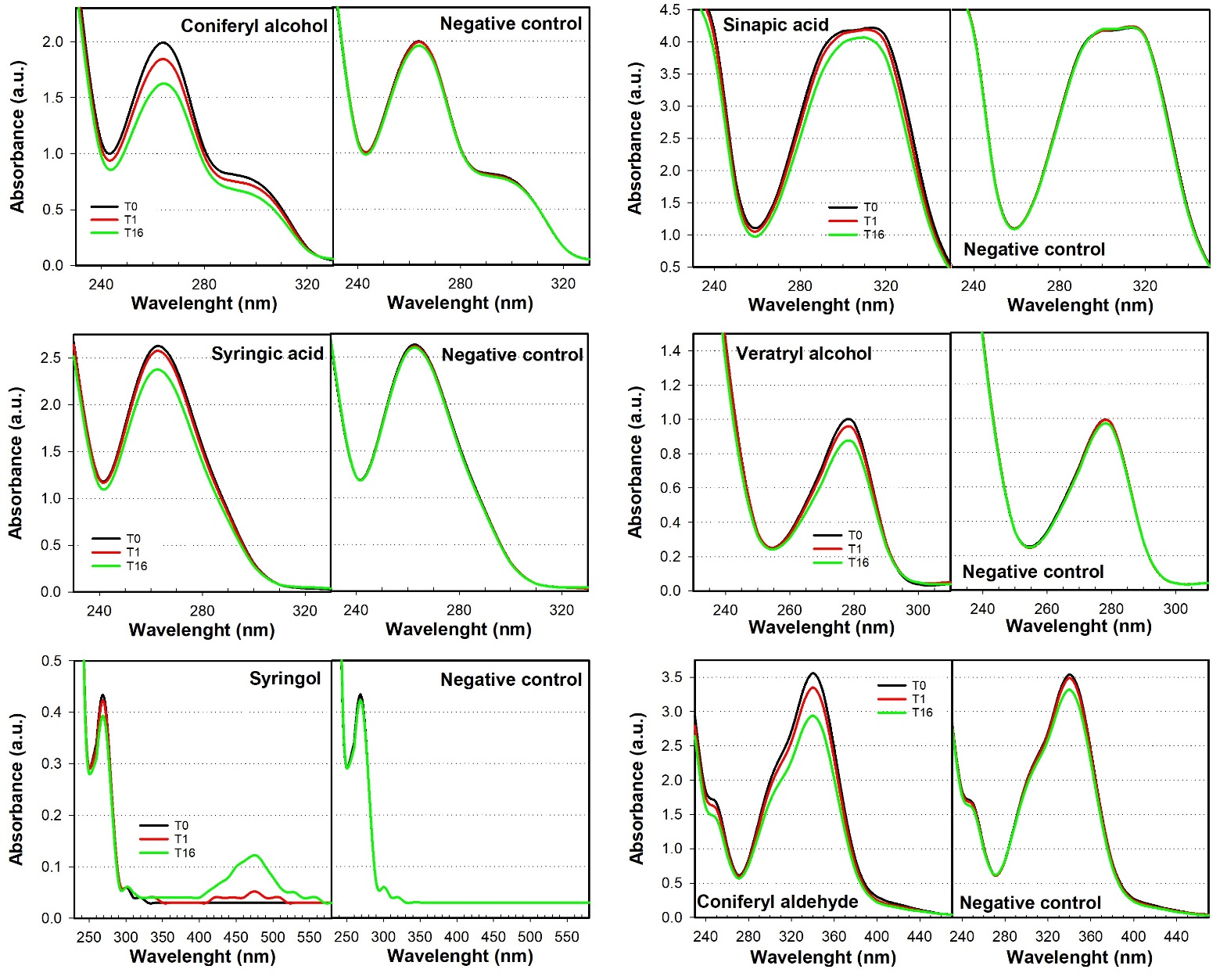** |
| **Fig. S15 –** The recorded absorbance spectrum of the reaction of *Hc*Tyr1 and the respective negative controls over time (T0 – starting time: T1 – 1 hour reaction; T16 – 16 hours reaction). The enzymatic reactions were carried out in a total volume of 250 µL containing 200 µL 20 mM MES pH 5.5, 25 µL *Hc*Tyr1 (0.05 mg/mL) (or the equivalent volume of water for the negative control) and 25 µL substrate (5 mM dissolved in 20 mM MES pH 5.5, 25 % (*v/v*) DMSO). |
| **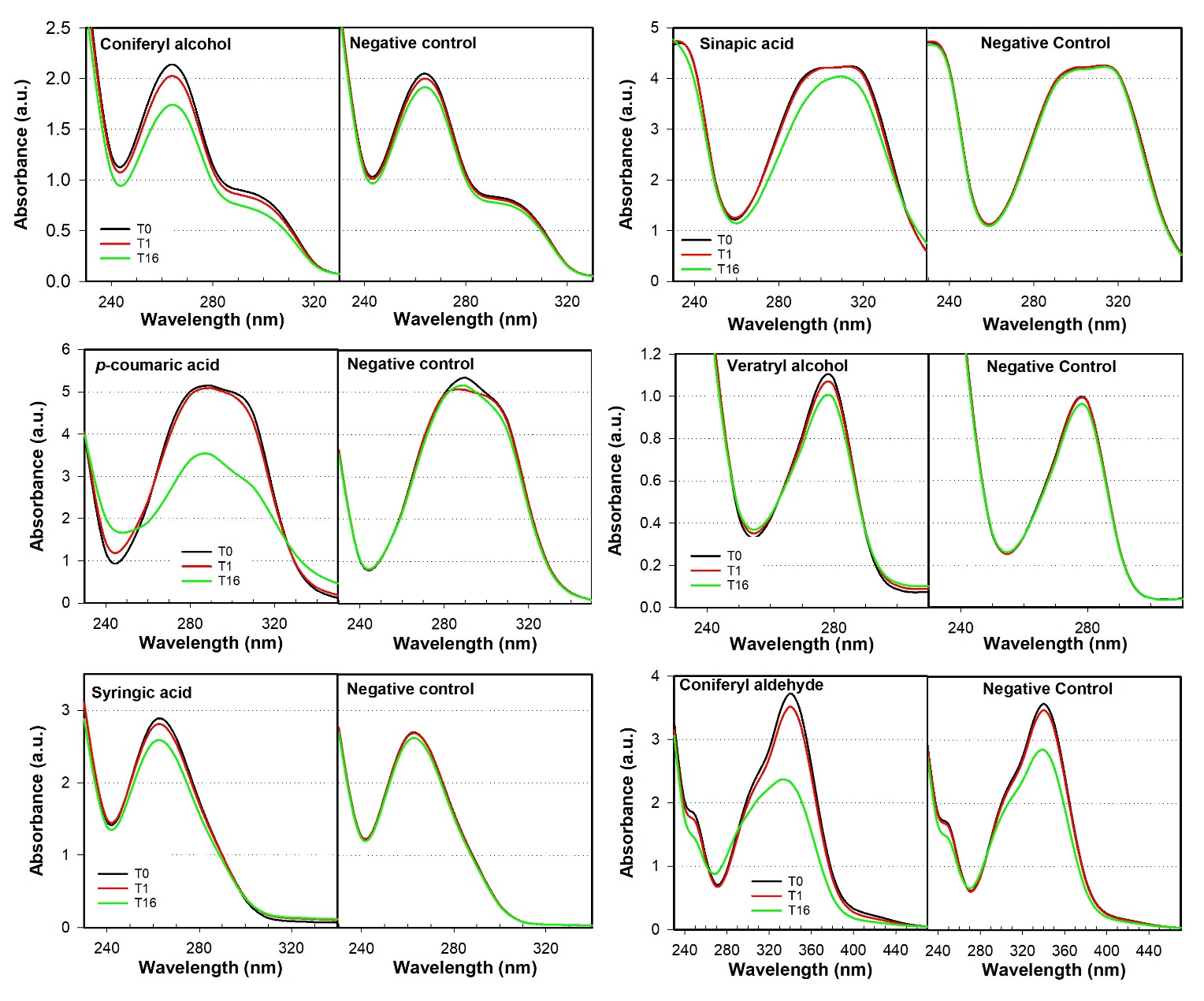** |
| **Fig. S16 –** The recorded absorbance spectrum of the reaction of *Hc*Tyr2 and the respective negative controls over time (T0 – starting time: T1 – 1 hour reaction; T16 – 16 hours reaction). The enzymatic reactions were carried out in a total volume of 250 µL containing 200 µL 20 mM MES pH 5.5, 25 µL *Hc*Tyr2 (0.3 mg/mL) (or the equivalent volume of water for the negative control) and 25 µL substrate (5 mM dissolved in 20 mM MES pH 5.5, 25 % (*v/v*) DMSO). |
| **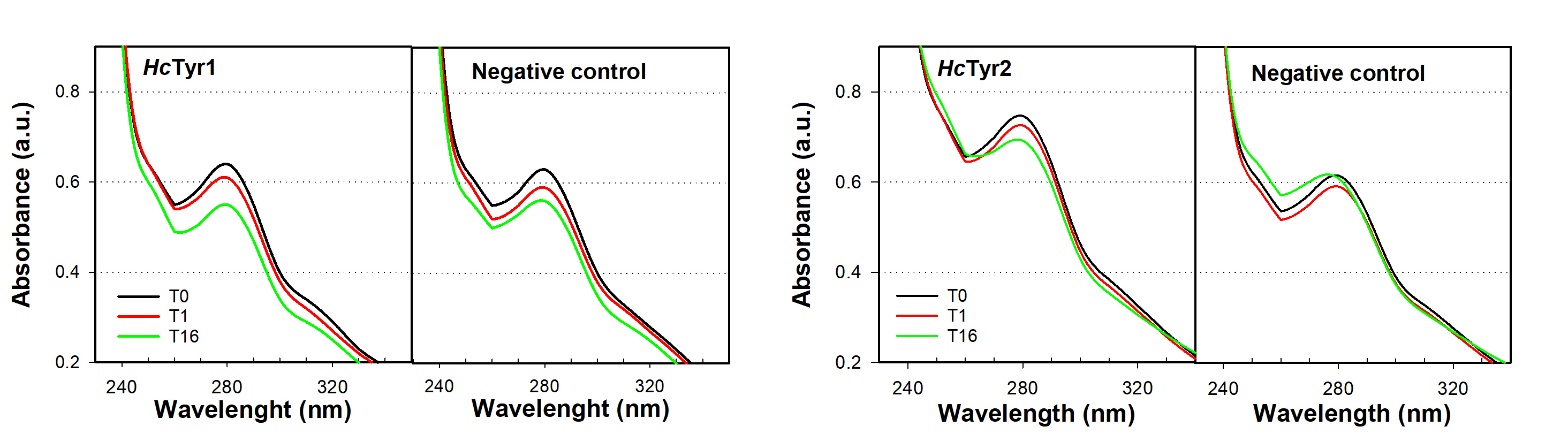** |
| **Fig. S17 –** The recorded absorbance spectrum of the reaction of *Hc*Tyr1 (left) and *Hc*Tyr2 (right) with lignin and the respective negative controls over time (T0 – starting time: T1 – 1 hour reaction; T16 – 16 hours reaction). The enzymatic reactions were carried out in a total volume of 250 µL containing 200 µL 20 mM MES pH 5.5, 25 µL *Hc*Tyr1 (0.05 mg/mL) or *Hc*Tyr2 (0.3 mg/mL) (or the equivalent volume of water for the negative control) and 25 µL substrate (lignin was added to 20 mM MES pH 5.5, 25 % (*v/v*) DMSO, sonicated to help dissolution, centrifuged and supernatant was used). |
| **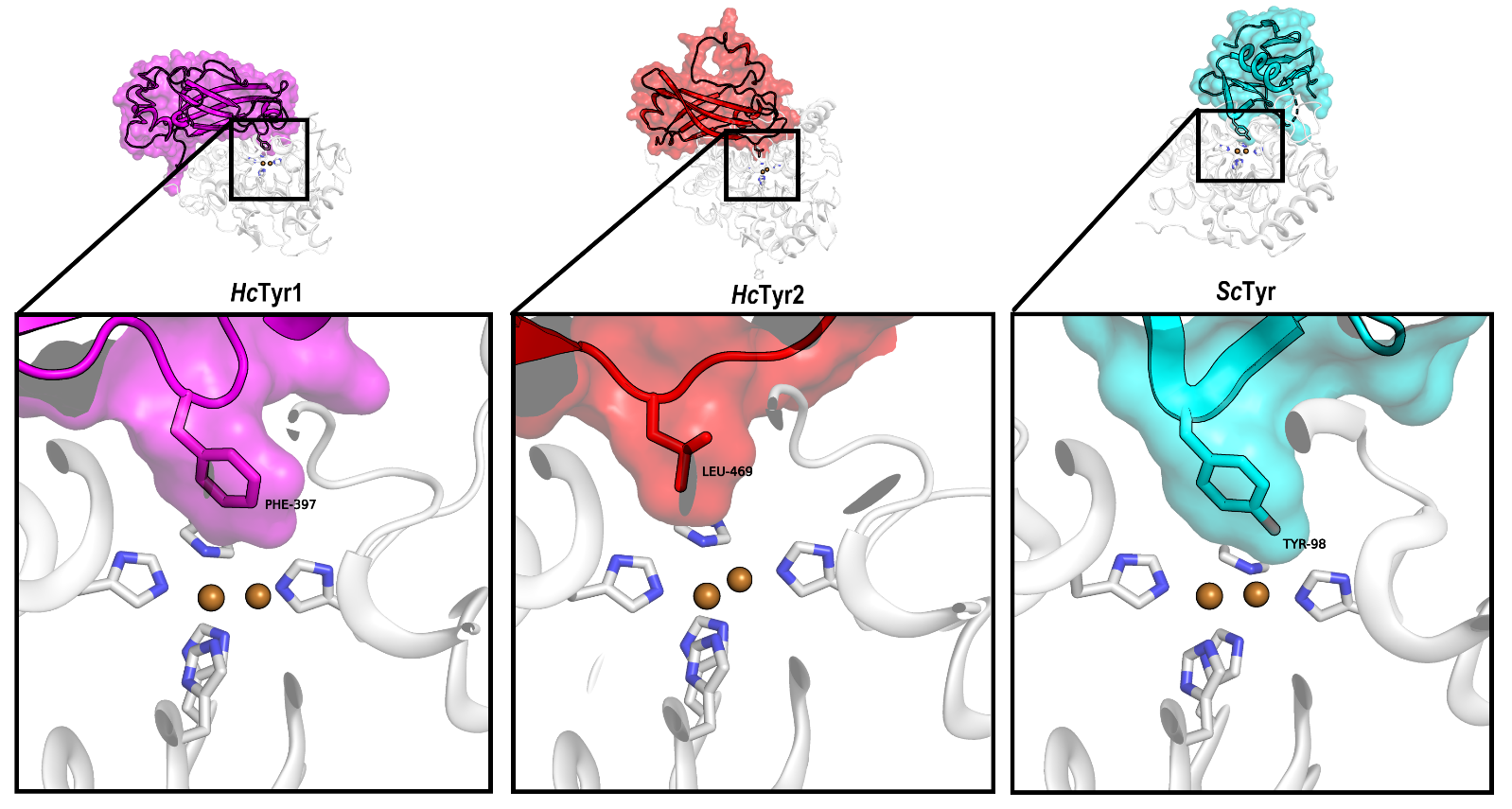** |
| **Fig. S18 –** The placeholder residues of *Hc*Tyr1, *Hc*Tyr2 (both AF2 models) and *Sc*Tyr (PDB: 1WX2). The LID-domains are depicted as a surface and cartoon, and the TYR-domains as cartoon. Placeholder residues and some of the histidines at the active site are represented as stick, and the two copper atoms are represented by brown spheres. Close-up views are also shown. The copper atoms in AF2 models are manually placed for illustration purposes. |
| **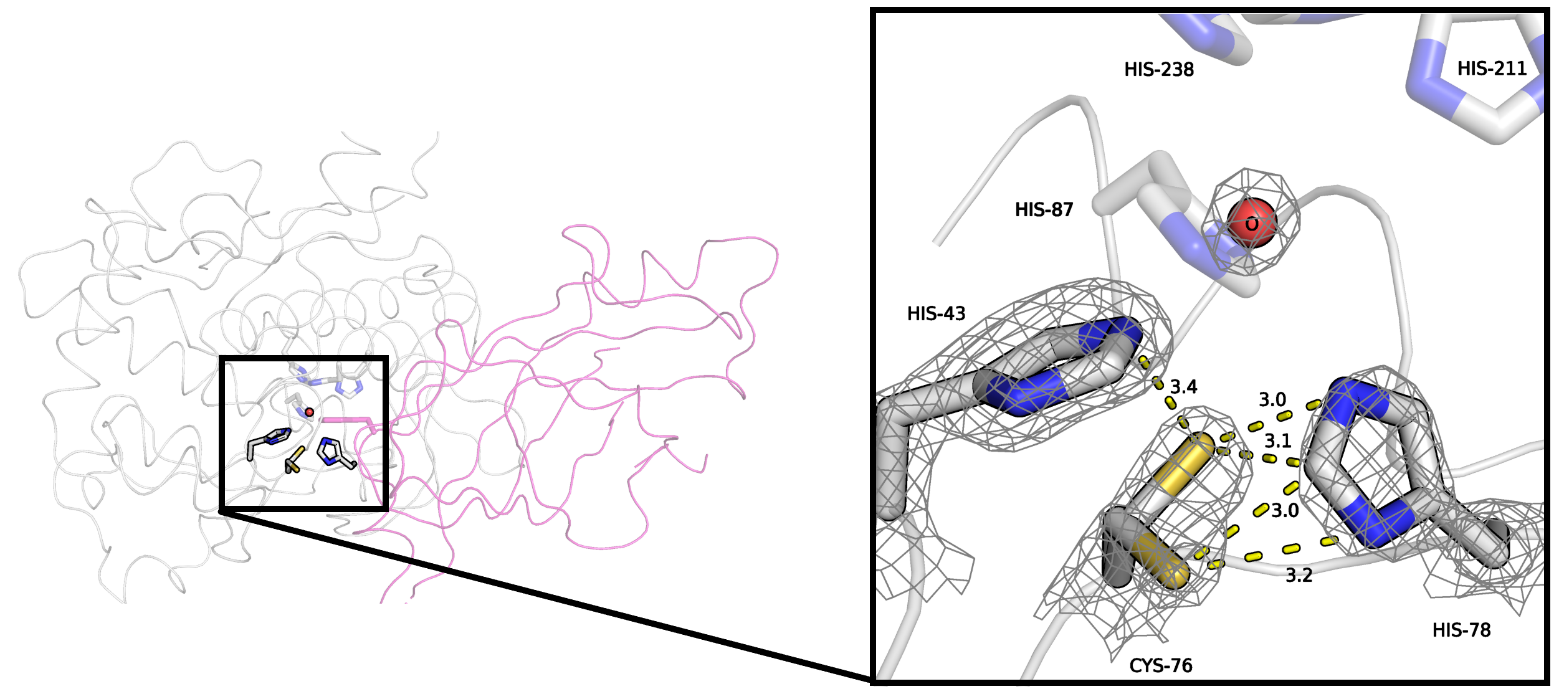** |
| **Fig. S19 –** The two Cys76 alternative conformations in HcTyr1 active site. Active site residues are depicted as sticks, oxygen from a water molecule is represented as a red sphere. Placeholder residues Phe397 is coloured magenta. 2Fo − Fc electron-density mesh (2σ) coloured in grey; |
| **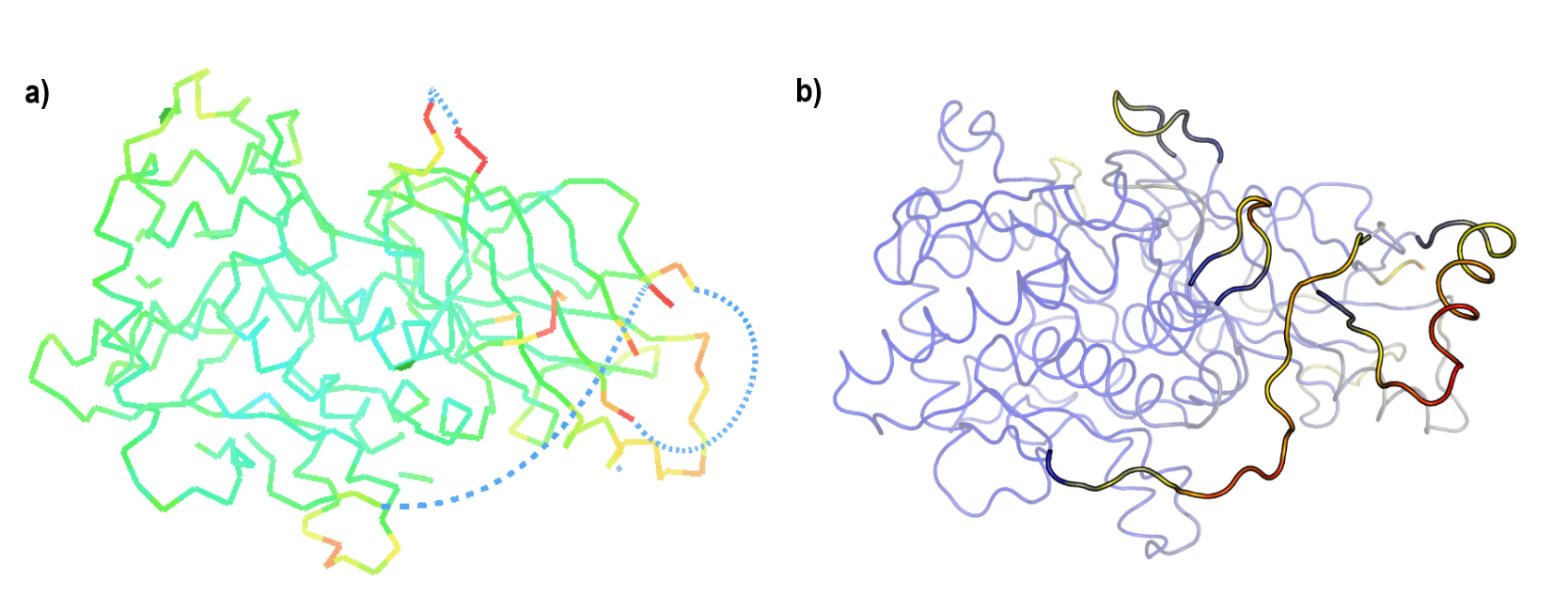** |
| **Fig. S20 –** The cartoon representation of *Hc*Tyr1 crystal structure (a) and its alphafold2 model (b) are shown side by side. Missing areas in “a” are depicted as a blue dashed line and highlighted in “b”. The b‑factors in (a) are spectrum coloured from green to yellow and then to red (representing low, medium and high, 0 to 100 b-factors, respectively.) and the per‑residue confidence score of AF2 in “b” are spectrum coloured from red to yellow and then to blue (representing low, medium and high, 0 to 100 per-residue confidence score, respectively). |

.

## Tables

|  |  | | | | | | | | | | | | |
| --- | --- | --- | --- | --- | --- | --- | --- | --- | --- | --- | --- | --- | --- |
| **Table S1 –** The relative activities of tyrosinases from different organisms in the presence of different metals at 1 mM. Activity without metal addition is taken as 100 %. | | | | | | | | | | | | | |
| **Source Org.** | | **Mn^2+^** | **Ni^2+^** | **Zn^2+^** | **Ca^2+^** | **Fe^2+^** | **Fe^3+^** | **Mg^2+^** | **Co^2+^** | **Al^3+^** | **Cu^2+^** | **Li^+^** | **Ref.** |
| ***Hahella* sp. CCB4 MM** | | 102±4 | 103±5 | 29±1 | 121±2 | n.d. | 0 | 102±1 | 99±3 | 71±4 | 33±2 | 111±5 | This work |
|  |  | 100±6 | 122±8 | 108±6 | 105±8 | n.d. | 0 | 105±8 | 107±6 | 18±2 | 45±1 | 98±8 |  |
| ***B. aryabhattai*** | | 88±1 | 73±1 | 45±1 | 90±1 | n.d. | 59±1 | 91±1 | 83±1 | n.d. | 52±2 | n.d. | [42] |
| ***A. ostoyae*** | | 125±10 | n.d. | 40±5 | 90 | 117±10 | n.d. | 95 | 66±8 | 111±8 | 37±3 | 90 | [95] |
| ***S. kathirae*** | | 120 | 57 | 98 | 81 | n.d. | 106 | 96 | 47 | 143 | 52 | n.d. | [58] |
| ***M. paradisiaca**** | | 118±0 | 115±1 | 103±1 | 80±1 | 194±2 | n.d. | 93±1 | 12±0 | n.d. | 138±0 | n.d. | [96] |
| ***M. mango*** | | 136 | n.d. | 134 | 197 | n.d. | n.d. | 128 | n.d. | n.d. | 33 | n.d. | [97] |
| ***D. polystachya*** | | 70 | n.d. | 80 | 50 | n.d. | 110 | 130 | n.d. | n.d. | 80 | n.d. | [98] |
| ***L. confusa*** | | 176±2 | n.d. | 106±2 | 107±2 | n.d. | n.d. | 122±2 | n.d. | n.d. | 48±2 | n.d. | [99] |
| ***S. polyantibioticus* SPRT** | | 106±2 | n.d. | 105±2 | 101±4 | 106±5 | n.d. | 114±2 | 111±2 | n.d. | 71±5 | n.d. | [76] |
| ***S. pharetrae* CZA14T** | | 99±4 | n.d. | 103±2 | 99±8 | 127±13 | n.d. | 109±6 | 118±3 | n.d. | 53±22 | n.d. | [76] |
| * All metals were tested at 0.5 mM  n.d.: not determined | |  |  |  |  |  |  |  |  |  |  |  |  |

| **Table S2** – The data collection and refinement statistics for recombinant *Hc*Tyr1.  Values in parentheses are for the outer shell. | | |
| --- | --- | --- |
| **Parameter** |  | **Value** |
| **X-Ray source** |  | ID‑30 (ESRF) |
| **Crystal Data** | | |
| Space group |  | P 2 2 2_1_ |
| Unit cell: a; b; c) |  | 51.14; 55.57; 156.3 (Å) |
| Unit cell: α; β; γ) |  | 90.0; 90.0; 90.0 ° |
| **Data Collection and processing** | | |
| Wavelength |  | 0.87313 Å |
| Resolution range |  | 78.27 - 1.97 (2.00 - 1.97) Å |
| Total number of observations |  | 119225 (5943) |
| Unique reflections |  | 32382 (1605) |
| R_merge_ |  | 0.081 (0.565) % |
| Mean (I/σ(I)) |  | 9.7 (2.3) |
| Completeness |  | 99.2 (100.0) % |
| CC_1/2_ |  | 0.997 (0.781) |
| Wilson B-factor |  | 29.1 Å^2^ |
| **Refinement statistics** |  |  |
| Reflections used in refinement |  | 32375 (3208) |
| Reflections used for R-free |  | 1701 (149) |
| R-work |  | 0.175 |
| R-free |  | 0.226 |
| Number of non-hydrogen atoms |  |  |
| Total |  | 3611 |
| Macromolecules |  | 3428 |
| Ligands |  | 0 |
| Solvent |  | 183 |
| Protein residues |  | 430 |
| RMS deviations |  |  |
| Bonds |  | 0.016 Å |
| Angles |  | 2.12 ° |
| Ramachandran plot |  |  |
| Favoured |  | 97.16 % |
| Allowed |  | 2.37% |
| Outliers |  | 0.47 % |
| Rotamer outliers |  | 6.81 % |
| Average B-factor |  |  |
| Overall |  | 35.6 Å^2^ |
| Macromolecules |  | 35.15 Å^2^ |
| Solvent |  | 35.67 Å^2^ |
